# Intra-Tumour Signalling Entropy Determines Clinical Outcome in Breast and Lung Cancer

**DOI:** 10.1371/journal.pcbi.1004115

**Published:** 2015-03-20

**Authors:** Christopher R. S. Banerji, Simone Severini, Carlos Caldas, Andrew E. Teschendorff

**Affiliations:** 1 Statistical Cancer Genomics, Paul O’Gorman Building, UCL Cancer Institute, University College London, London WC1E 6BT, UK; 2 Department of Computer Science, University College London, London WC1E 6BT, UK; 3 Centre of Mathematics and Physics in the Life Sciences and Experimental Biology, University College London, London WC1E 6BT, UK; 4 Breast Cancer Functional Genomics Laboratory, Cancer Research UK, Cambridge Institute, University of Cambridge, Li Ka Shing Centre, Robinson Way, Cambridge, UK; 5 CAS-MPG Partner Institute for Computational Biology, Chinese Academy of Sciences, Shanghai Institute for Biological Sciences, 320 Yue Yang Road, Shanghai 200031, China; Weizmann Institute of Science, ISRAEL

## Abstract

The cancer stem cell hypothesis, that a small population of tumour cells are responsible for tumorigenesis and cancer progression, is becoming widely accepted and recent evidence has suggested a prognostic and predictive role for such cells. Intra-tumour heterogeneity, the diversity of the cancer cell population within the tumour of an individual patient, is related to cancer stem cells and is also considered a potential prognostic indicator in oncology. The measurement of cancer stem cell abundance and intra-tumour heterogeneity in a clinically relevant manner however, currently presents a challenge. Here we propose signalling entropy, a measure of signalling pathway promiscuity derived from a sample’s genome-wide gene expression profile, as an estimate of the stemness of a tumour sample. By considering over 500 mixtures of diverse cellular expression profiles, we reveal that signalling entropy also associates with intra-tumour heterogeneity. By analysing 3668 breast cancer and 1692 lung adenocarcinoma samples, we further demonstrate that signalling entropy correlates negatively with survival, outperforming leading clinical gene expression based prognostic tools. Signalling entropy is found to be a general prognostic measure, valid in different breast cancer clinical subgroups, as well as within *stage I* lung adenocarcinoma. We find that its prognostic power is driven by genes involved in cancer stem cells and treatment resistance. In summary, by approximating both stemness and intra-tumour heterogeneity, signalling entropy provides a powerful prognostic measure across different epithelial cancers.

## Author Summary

The Cancer Stem Cell (CSC) hypothesis, the idea that a small population of tumour cells have the capacity to seed and grow the tumour, and intra-tumour heterogeneity, the diversity of the cancer cell population within the tumour of an individual patient, have long been considered the basis of potential prognostic indicators in oncology. The identification of CSC based expression signatures and the measurement of intra-tumour heterogeneity, for an assessment of prognostic power in a clinically relevant manner, however, currently presents a challenge. Most proposed methodologies require the collection of new data sets and thus are limited in sample size, making them difficult to validate. Here we consider signalling entropy, a measure of signalling pathway promiscuity, as a means of quantifying the stemness and heterogeneity of any given cancer sample, applicable to publicly available data sets. By considering over 5300 primary tumour samples from both breast and lung cancer patients, we here demonstrate that signalling entropy provides a more robust and general prognostic measure than other leading clinical prognostic indicators.

## Introduction

Over recent years considerable evidence has arisen supporting the hypothesis that some cancers are hierarchically organised, akin to the organisation of healthy cells, with a small population of Cancer Stem Cells (CSCs) driving a heterogeneous, hierarchical structure [1, 2]. The abundance of CSCs is considered likely to be of prognostic value as well as a source of intra-tumour heterogeneity, a feature that has long been considered of possible prognostic value in oncology [3–6]. Although putative CSCs have been identified by surface marker expression for several malignancies, isolated, and demonstrated to be chemotherapeutic resistant [7–11], it remains a significant challenge to obtain a prognostic measure of their abundance from tumour bulk gene expression profiles across multiple malignancies. Embryonic Stem (ES) cell gene expression signatures are clear candidates for such a measure and indeed have been demonstrated to be prognostic in breast and lung cancer [12–15]. Their overall prognostic significance seems limited, however, and they are unable to discriminate CSCs from the tumour bulk [12, 16]. The clinical assessment of intra-tumour heterogeneity also poses a significant challenge, with current experimental approaches requiring multiple biopsies per tumour leaving them severely limited in sample size [17–19]. We posited that an expression based measure of signalling promiscuity may quantify the stemness of a tumour in a manner which is related to intra-tumour heterogeneity, and thus provide us with an improved prognostic model.

Here we explore this hypothesis, using an *in-silico* approach. Specifically, we consider *signalling entropy* which is computed from the integration of a sample’s genome-wide gene expression profile with an interactome, and provides an overall measure of the signalling promiscuity in the sample [16]. We note that the term signalling entropy was chosen, as opposed to alternatives such as interactome/network entropy, to emphasise the fact that our measure quantifies network traffic (signalling) as opposed to network topology. Importantly, as shown by us previously, signalling entropy correlates with stemness and differentiation potential within distinct cellular developmental lineages [16]. Indeed, we showed that human embryonic stem cells and induced pluripotent stem cells exhibited the highest levels of signalling entropy, with adult stem cells (e.g. hematopoietic stem cells) showing significantly lower values, and terminally differentiated cells exhibiting the lowest entropy values within a lineage [16]. These results were derived mostly from cell-lines, which are characterised by relatively homogeneous cell populations, and were further validated in time-course differentiation experiments [16]. Importantly, we also demonstrated that cancerous tissue displays a higher signalling entropy than its healthy counterpart [16, 20], with CSCs showing higher values than the tumour bulk [16]. Thus, signalling entropy provides an approximation of the stemness of a cellular sample.

In addition to quantifying stemness of the signalling regime of a homogeneous cell population, signalling entropy, if computed over a heterogeneous cell population, should also quantify the inter-cellular diversity in pathway activation. To investigate this we performed an analytical investigation of signalling entropy, coupled with empirical validation. We derived a sufficient condition on the expression profiles of homogeneous cell populations for signalling entropy to be a measure of intra-sample heterogeneity on average. We subsequently verified that this condition is satisfied by considering 33 distinct adult tissue expression profiles corresponding to 528 pairwise mixtures. Thus, we show that signalling entropy is a good candidate for a correlate of intra-sample heterogeneity.

Importantly, because signalling entropy can be computed from a bulk tumour gene expression profile, it allows us to assess the prognostic significance of our measure in large numbers of clinical specimens. We here compute signalling entropy for a total of 5360 tumour samples, focusing on two highly heterogeneous cancers, non-small cell lung cancer (NSCLC) and breast cancer, which constitute the two leading causes of cancer death world-wide [21]. Survival rates for early stage NSCLC are particularly poor [21, 22], and identification of prognostic and predictive biomarkers within the *stage I* stratum is considered a high priority [23]. In breast cancer, the power of gene expression based prognostic indicators, such as OncotypeDX and MammaPrint [24, 25], is highly subtype dependent [26, 27] and a clinical breast cancer prognostic signature, which is independent of estrogen receptor (ER) status is lacking. Most importantly, current gene expression based prognostic indicators ignore CSC contributions and intra-tumour heterogeneity [17]. Thus, signalling entropy, a measure of both cell anaplasia and intra-tumour heterogeneity, may form the basis of a general and more robust prognostic indicator. By examining gene expression profiles of over 3500 primary breast cancers and 1300 lung adenocarcinomas, we here demonstrate that signalling entropy is prognostic in breast cancer, regardless of ER status, and in lung adenocarcinomas, within the *stage I* stratum.

## Results

### Rationale of signalling entropy as a prognostic measure

Signalling entropy is derived from the integration of a sample’s gene expression profile with a human protein interactome, and provides a rough proxy for the overall level of signalling promiscuity in the sample. Briefly, we employ the mass-action principle to derive, for each sample, a stochastic matrix *p*_*ij*_, describing the interaction probability of the proteins encoded by genes *i* and *j* in the given sample. The signalling entropy is then computed as the normalised entropy rate of the Markov chain described by *p*_*ij*_. This entropy rate gives a steady state measure of the disorder (or promiscuity) in signalling information flow over the network in the given sample (Materials and Methods).

**Fig 1:**
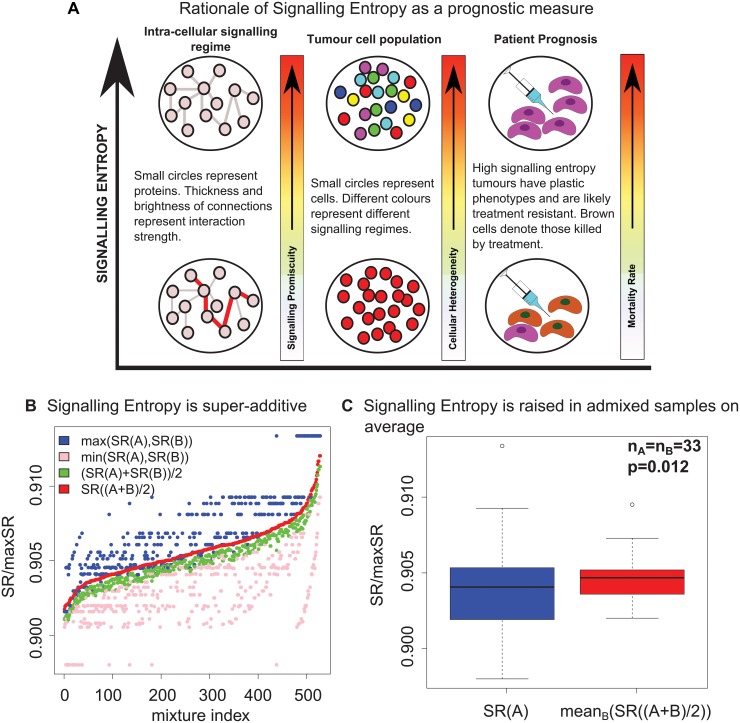
Rationale behind signalling entropy as a prognostic factor in cancer. A) A high signalling entropy of a tumour sample indicates a promiscuous, stem cell like intra-cellular signalling regime and a heterogeneous cancer cell population. The consequence of a high entropy is thus a tumour with a plastic phenotype, capable of activating diverse pathways in response to treatment. High signalling entropy tumours are thus likely to result in higher patient mortality. B) Signalling entropy (denoted SR/max SR) computed for 528 distinct pairwise mixtures of 33 homogeneous tissue samples reveals that our measure is super-additive and hence will be raised, on average, in mixed samples compared to homogeneous samples. C) Signalling entropy is raised on average in mixed samples as compared to homogeneous samples, considering the same 33 homogeneous tissue samples as Fig. 1B. The *p*-value corresponds to a two tailed paired Wilcoxon signed rank test, and reveals that signalling entropy is significantly elevated in the admixed cell populations on average.

As shown by us previously, stem cells have a high signalling entropy which decreases during differentiation, a result not forthcoming using other molecular entropy measures [16, 28]. Importantly, we also demonstrated that signalling entropy is elevated in CSCs as compared to the tumour bulk [16]. Thus, given a homogeneous cell population, a high signalling entropy suggests that signalling within each cell is very promiscuous and that the cells may therefore have a plastic stem cell like phenotype. However, a heterogeneous sample, consisting of cells with distinct, though not necessarily promiscuous signalling regimes, should also on average display a high signalling entropy, suggesting that signalling entropy may associate with intra-tumour heterogeneity (Fig. 1A).

To investigate whether signalling entropy associates with intra-sample heterogeneity, we considered our measure evaluated for three theoretical samples: namely two homogeneous samples consisting only of cell type *x* or *y* respectively, and a third heterogeneous sample consisting of a 50:50 mixture of cell types *x* and *y*. It is clear that if cell type *x* has an expression profile that maximises signalling entropy and cell type *y* does not, then the signalling entropy of the mixture will be lower than the signalling entropy of *x*, thus signalling entropy is not a point-wise measure of heterogeneity. However, as most biologically realistic cell types have distinct expression profiles, corresponding to the existence of non-overlapping active pathways between cell type pairs [29], we posited that the signalling entropy of a mixed sample may be higher than that of a homogeneous sample on average.

By appealing to detailed balance we examined a closed form expression for signalling entropy. It is a consequence of simple algebra that if signalling entropy is super-additive over the set of biologically admissible expression profiles (*i.e*., Signalling Entropy $\left(\frac{x+y}{2}\right)>$ $\frac{1}{2}$ Signalling Entropy $(x)+\frac{1}{2}$ Signalling Entropy(*y*)) then signalling entropy will on average be elevated in mixed samples as opposed to homogeneous samples (Materials and Methods, S1 Text, S8 Fig, S9 Fig, S10 Fig and S11 Fig). We thus derived a condition for point-wise super-additivity of our measure and then considered a data set of gene expression profiles for 33 distinct adult tissues, representing 528 possible pairwise mixtures [29]. For every possible mixture the derived condition for super-additivity was satisfied (Fig. 1B). Whence the signalling entropies of the mixed samples was significantly higher than that of homogeneous samples on average (Fig. 1C). This provides strong evidence that signalling entropy is a correlate of intra-sample heterogeneity.

Thus, signalling entropy associates with tumour stemness in a manner associated with CSC abundance and intra-tumour heterogeneity, making our measure a good candidate for an improved prognostic indicator.

### Signalling entropy is prognostic in the major subtypes of breast cancer

In order to assess the prognostic significance of signalling entropy in breast cancer, we first computed its value for each microarray sample of the Molecular Taxonomy of Breast Cancer International Research Consortium dataset (METABRIC) [30], a total of 1980 samples divided into a discovery and validation sets of equal proportion. This data set profiles a large number of clinical variables and thus is a suitable platform to examine the clinical associations of our measure. Using outcome first as a binary phenotype, we observed that patients who died of breast cancer had a higher signalling entropy than patients who were alive at last follow up, a result which was seen in both METABRIC subsets (*p* < 1*e* − 7). Using a Cox proportional hazards model, on 5 year censored survival data, we ascertained that high signalling entropy is associated with increased risk of death in breast cancer (c-index = 0.6, *p* < 1.1*e* − 6). Stratifying patients into 3 groups, representing the 3 tertiles of the signalling entropy distribution, revealed that tumours with a high entropy exhibited a doubling of the hazard rate compared to low entropy tumours.

Signalling entropy was found to be associated with tumour grade and ER status, however, its prognostic power was independent of these variables, as well as of stage, p53 status, tumour size and lymph node status (S1 Text, S1 Fig & S2 Fig). In addition, signalling entropy was also found to be independent of a prognostic ES cell signature described by Ben-Porath *el al*. [12] and the prognostic grade signature described by Sotiriou *et al*. [31] (S1 Text, S1 Fig & S2 Fig). Signalling entropy was significantly prognostic within each tumour grade strata; notably it was prognostic within the grade 2 stratum in both METABRIC data sets (*p* < 0.036), an important result given the difficulty in deciding treatment courses in this intermediate prognosis group [31]. The fact that signalling entropy is prognostic independently of all other measures of cell anaplasia, suggests that our measure may be capturing more than just the stemness of a tumour sample, and that intra-tumour heterogeneity may be contributing to its prognostic power.

A recent study by Venet *et al*. described prognostic associations for a number of random gene expression signatures in breast cancer [32]. To ascertain whether random effects may be driving our findings, we evaluated the prognostic associations of the three random gene expression signatures described by Venet *et al*.. We found that only one was prognostic in both discovery and validation METABRIC data sets and that its prognostic power was determined by ER status (S3 Fig). To further assess the impact of random effects and the importance of our network, we randomised the gene expression profiles of the METABRIC data sets over the network. Performing 5 randomisations and recomputing signalling entropy for the 1980 samples in both METABRIC data sets, revealed that randomised signalling entropy did not display robust prognostic associations independently of ER status. We are therefore confident that the prognostic power of signalling entropy is not driven by random effects.

**Fig 2:**
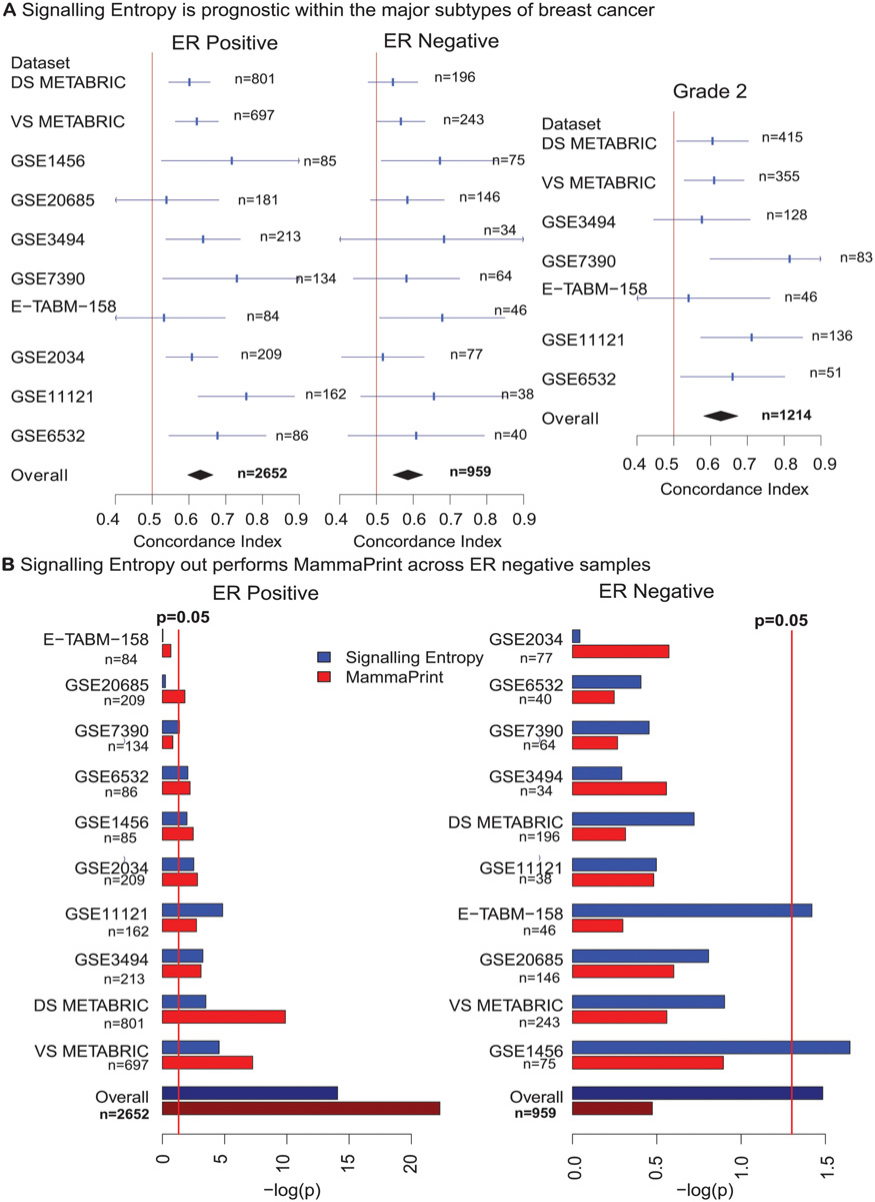
Prognostic implications of signalling entropy in breast cancer. A) The plots display the concordance index for signalling entropy in each data set alongside its 95% confidence interval. The overall concordance index was derived via meta-analysis using a random effects model. The vertical line denotes concordance index = 0.5, data sets where the confidence interval for the concordance index crosses this line did not reach significance. Meta-analysis of signalling entropy across 10 breast cancer data sets reveals that our measure is significantly prognostic across both ER positive and ER negative subtypes. Meta-analysis across 7 breast cancer data sets reveals that our measure is also significantly prognostic within the grade 2 stratum. B) The plots display the negative of the log_10_ of the *p*-value for a survival analysis using Cox-regression on 5-year censored data, evaluating the prognostic significance of signalling entropy and MammaPrint in each data set. The overall *p*-value was produced by a Fisher’s combined test. The vertical red line on each plot denotes *p* = 0.05; data sets in which the bar crosses this line reached significance for the corresponding score. Meta-analysis comparison of signalling entropy with MammaPrint across 10 breast cancer data sets, demonstrates that only signalling entropy is significantly prognostic across ER negative samples.

To further validate the prognostic impact of signalling entropy we considered eight further independent breast cancer data sets. All these datasets described both ER positive and negative tumours with accompanying clinical outcome, profiled on either Affymetrix or Illummina platforms and totalling 1688 samples [33–40], (S1 Table). Meta-analysis revealed that signalling entropy is prognostic across both ER positive and ER negative samples (ER positive: c-index = 0.63, 95% CI = (0.604, 0.657), *p* = 8.5*e* − 15, ER negative: c-index = 0.57, 95% CI = (0.538, 0.602), *p* = 0.032, Fig. 2A). Five of the additional eight data sets also described histological tumour grade for each sample, allowing us to further confirm that signalling entropy is prognostic within the grade 2 stratum (c-index = 0.63, 95% CI = (0.581, 0.675), *p* = 1.05*e* − 6, Fig. 2A).

These results are in contrast to the performance of MammaPrint, a microarray based breast cancer prognostic signature currently being assessed in the MINDACT trial [41]. In a meta-analysis over the 10 breast cancer validation sets we found that unlike signalling entropy MammaPrint was not significantly prognostic over ER negative samples (Fig. 2B).

Another popular breast cancer prognostic assay in clinical trials is OncotypeDX, which uses RT-PCR to quantify the expression of genes associated with survival [25]. Due to differences in the normalisation between RT-PCR and microarrays, a direct comparison between our measure and OncotypeDX is difficult to perform. Moreover, not all the genes required for computing the OncotypeDX recurrence score were present in all the array platforms considered. However, using a microarray version of OncotypeDX, we found that it performed comparably to signalling entropy across both ER positive (signalling entropy vs. OncotypeDX: *p* = 0.13) and ER negative samples (signalling entropy vs. OncotypeDX: *p* = 0.7, S4 Fig).

Thus signalling entropy is prognostic in the two major clinical subtypes of breast cancer and hence is a more robust prognostic indicator than MammaPrint.

### Signalling entropy is prognostic in *stage I* lung adenocarcinoma

We next investigated the prognostic power of our measure in lung adenocarcinoma. To evaluate the clinical associations of our measure we first computed signalling entropy for each microarray sample in The Director’s Challenge dataset profiling 398 tumours [42], and for the 455 lung adenocarcinoma RNA-seq tumour samples downloaded from The Cancer Genome Atlas (TCGA) database (http://cancergenome.nih.gov/). We found that signalling entropy was significantly lower in lung adenocarcinoma patients who were alive at last follow up as opposed to those who had died (*p* < 0.03). Fitting Cox proportional hazard models to 3 year censored data revealed that an increased signalling entropy implied a worse prognosis in lung adenocarcinoma (c-index = 0.6, *p* < 0.007). We again separated patients into tertiles of the signalling entropy distribution and found that high signalling entropy conferred almost a doubling of the hazard rate, as assessed over the first 3 years following diagnosis (HR = 1.9, *p* < 0.02).

Signalling entropy was found to be associated with tumour stage, grade and smoking status, in both TCGA and Director’s Challenge data sets, yet importantly the prognostic power of signalling entropy was independent of these clinical variables (S1 Text, S5 Fig & S6 Fig). It is of particular note that signalling entropy is significantly prognostic if computed from either microarray or RNA-seq data sets, this result attests to the biological relevance of our measure which is not masked by experimental technique.

**Fig 3:**
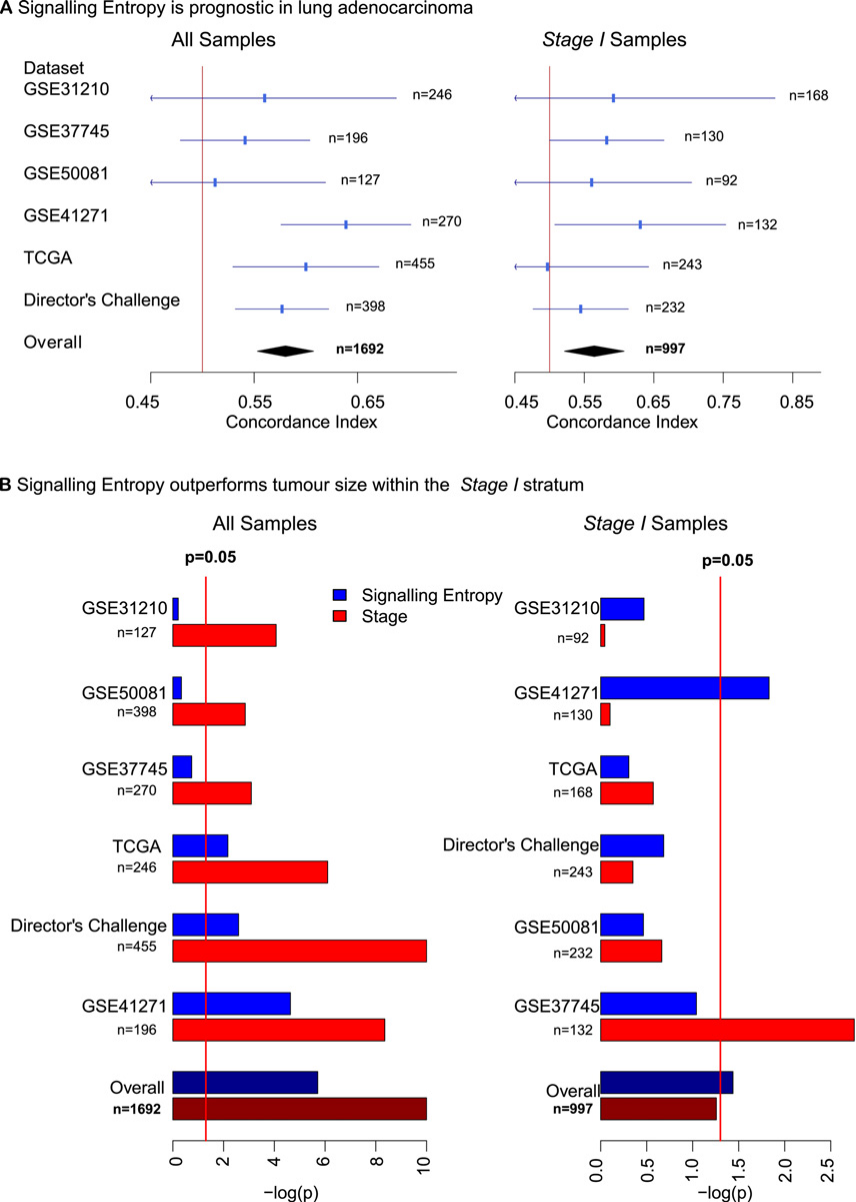
Prognostic implications of signalling entropy in lung adenocarcinoma. A) The plots display the concordance index for signalling entropy in each data set alongside its 95% confidence interval. The overall concordance index was derived via meta-analysis using a random effects model. The vertical line denotes concordance index = 0.5, data sets where the confidence interval for the concordance index crosses this line did not reach significance. Meta-analysis of signalling entropy across 7 lung adenocarcinoma data sets reveals that our measure is significantly prognostic across all samples and within the *stage I* stratum. B) The plots display the negative of the log_10_ of the *p*-value for a survival analysis using Cox-regression on 3-year censored data, evaluating the prognostic significance of signalling entropy and tumour stage in each data set. The overall *p*-value was produced by a Fisher’s combined test. The vertical red line on each plot denotes *p* = 0.05; data sets in which the bar crosses this line reached significance for the corresponding score. Meta-analysis comparison of signalling entropy with pathological tumour stage across 7 lung adenocarcinoma data sets, demonstrates that signalling entropy outperforms the *stage Ia/b* sub staging across *stage I* samples.

To validate the prognostic power of signalling entropy in lung adenocarcinoma, we performed a meta-analysis across 4 further independent data sets consisting of a total of 522 lung adenocarcinomas (S2 Table) [43–46]. This revealed that signalling entropy is prognostic across all samples and across *stage I* samples (all samples: c-index = 0.58, 95% CI = (0.55, 0.60), *p* = 1.9*e* − 6, *stage I*: c-index = 0.56, 95% CI = (0.52, 0.60), *p* = 0.037, Fig. 3A).

Early stage lung adenocarcinoma suffers from a high relapse rate and it is important to establish more robust prognostic assessments in the *stage I* subgroup for chemotherapeutic treatment stratification [22]. Sub-staging by size is currently the standard clinical approach to stratify *stage I* tumours, however, on meta-analysis we found that this stratification, unlike signalling entropy was not significantly prognostic over the *stage I* stratum (Fig. 3B).

### Signalling entropy’s prognostic power in breast cancer can be represented by a small number of genes

Signalling entropy is a clear prognostic indicator in breast cancer, yet its computation requires the expression of many thousands of genes, something which is currently cumbersome and expensive for clinical application. Moreover, our measure associates with tumour grade and ER status in breast cancer and thus the factors driving its prognostic power independently of these variables is unclear. We posited that the prognostic power of our measure, independent of ER status and grade may be captured by the expression of a small number of genes, analogously to the way the prognostic power of tumour grade was captured by the expression of the 97 gene Sotiriou *et al*. signature [31].

To identify suitable genes representative of signalling entropy’s prognostic power, we first investigated prognostic genes, which were correlated or anti-correlated with signalling entropy independently of grade and ER status, and whose prognostic power was also independent of grade and ER status. We then refined this gene set by fitting a Cox proportional hazards model on 5 year censored data using all the identified genes as covariates and deleting genes which were not significantly prognostic independently of others in the gene set. This resulted in a small set of 81 genes, 10 of which were negatively correlated with signalling entropy and 71 of which were positively correlated S2 Table. A Signalling Entropy prognostic score (SE score) was then defined as the *t*-statistic evaluating the hypothesis that the 71 positively correlated genes are expressed more highly than the 10 negatively correlated genes (after *z*-score normalising the data).

By using signalling entropy to refine a set of prognostic genes identified by Cox regression, our approach refines the feature selection approach based on correlation with outcome [24]. Consequently, the genes utilised to construct our SE score are both correlated with outcome and with signalling entropy and thus should provide a prognostic indicator representative of signalling promiscuity. Criticism of feature selection for prognostic classifiers based on gene sets ranked by correlation with outcome has stemmed from the considerable discordance of such features between data sets [47, 48]. By using signalling entropy to refine the prognostic gene set we found that this gene set instability was reduced. The genes which were both prognostic and correlated with signalling entropy showed more concordance between discovery and validation sets of METABRIC as compared to the genes which were only prognostic. Moreover, this increase in overlap was significantly higher than would be expected by chance (*p* < 10*e* − 5, based on re-sampling size matched sets of prognostic genes and assessing overlap). To further confirm this increased rodustness, we derived a set of genes for constructing an SE score from the METABRIC validation set, using an identical procedure to that performed on the discovery set. This gene list was slightly shorter than for the discovery set (55 genes, 34 positively correlated and 13 negatively correlated with signalling entropy) but had an overlap of 4 genes, significantly more than would be expected by chance (*p* = 0.012, based on re-sampling size matched sets of prognostic genes and assessing overlap). We provide the lists of prognostic genes both correlated and uncorrelated with signalling entropy as well as the validation set derived SE score genes in S3 Table.

**Fig 4:**
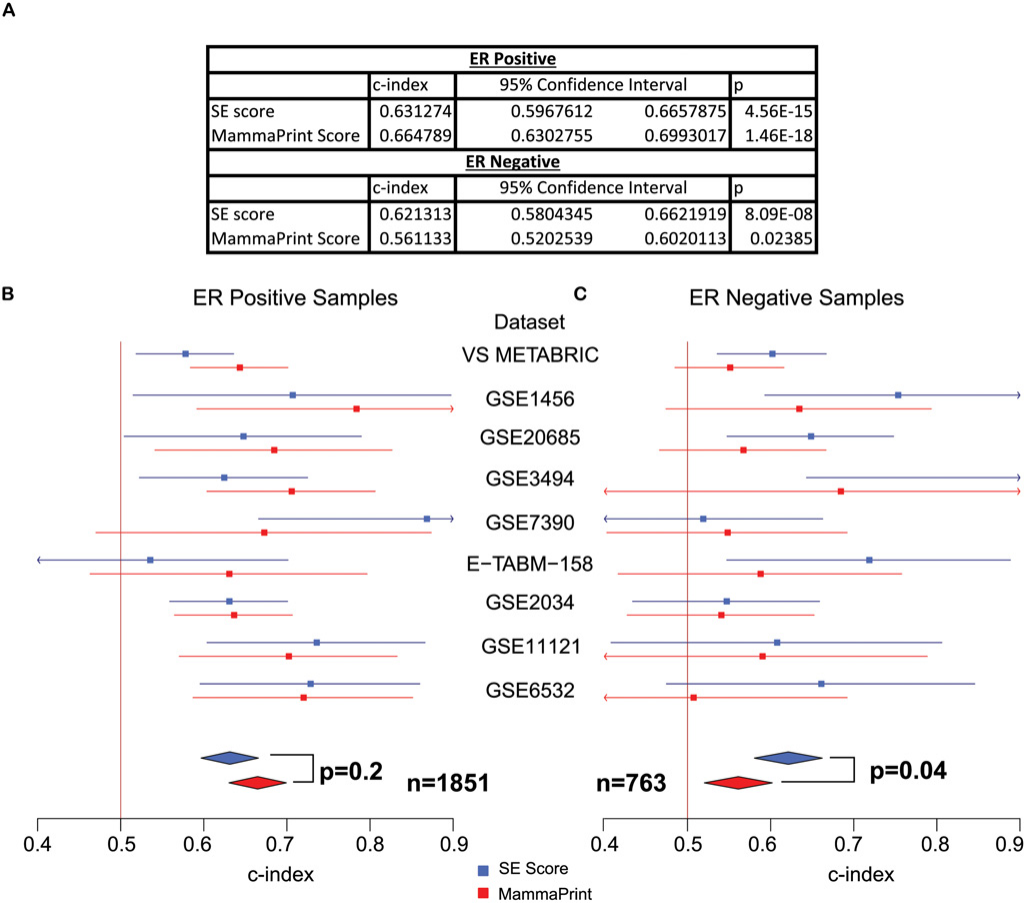
Meta-analysis comparison of the breast cancer SE score with MammaPrint. A) Survival analysis statistics for the SE score and MammaPrint over ER positive samples and ER negative samples separately, c-index denotes concordance index and p denotes *p*-value. B) & C) The plots display the concordance index for the SE score and MammaPrint in each data set alongside 95% confidence intervals. The overall concordance indices were derived and compared via meta-analysis using a random effects model. The vertical line denotes concordance index = 0.5, data sets where the confidence interval for the concordance index crosses this line did not reach significance. Meta-analysis reveals that the SE score performs comparably to MammaPrint in ER positive samples (B) and outperforms MammaPrint across ER negative samples (C).

Meta-analysis across 9 independent breast cancer data sets revealed that like signalling entropy, the SE score is prognostic across both ER positive and ER negative samples (ER positive: c-index = 0.63, 95% CI = (0.59, 0.67), *p* = 4.6*e* − 15, ER negative: c-index = 0.62, 95% CI = (0.58, 0.66), *p* = 8.1*e* − 8, Fig. 4A). Moreover, meta-analysis further demonstrated that the SE score performed comparably to MammaPrint over ER positive samples (SE score vs MammaPrint: *p* = 0.18, Fig. 4B), and out-performed MammaPrint over ER negative samples (SE score vs MammaPrint: *p* = 0.04, Fig. 4C). Whence the prognostic power of our measure is well captured by the expression of this small set of genes.

### A signalling entropy derived prognostic score outperforms microarray based prognostic indicators in lung adenocarcinoma

We next investigated whether a similar SE score could be computed for lung adenocarcinoma. Signalling entropy is correlated with, yet prognostically independent of tumour stage in lung adenocarcinoma, we therefore aimed to derive a score that represented the prognostic power of our measure independently of tumour stage. To achieve this we considered the Director’s Challenge data set of 398 lung adenocarcinomas as a discovery set [42]. We performed an analogous procedure as described above for breast cancer to identify genes associated with signalling entropy’s prognostic power independently of tumour stage in lung cancer, with the only differences being that we adjusted for tumour stage, rather than ER status and grade, and used 3 year censored data rather than 5 year. This resulted in a small set of 29 genes, 8 of which were negatively correlated with signalling entropy and 21 of which were positively correlated (S4 Table). An SE score was then defined again as the *t*-statistic evaluating the hypothesis that the positively correlated genes are expressed more highly than the negative.

**Fig 5:**
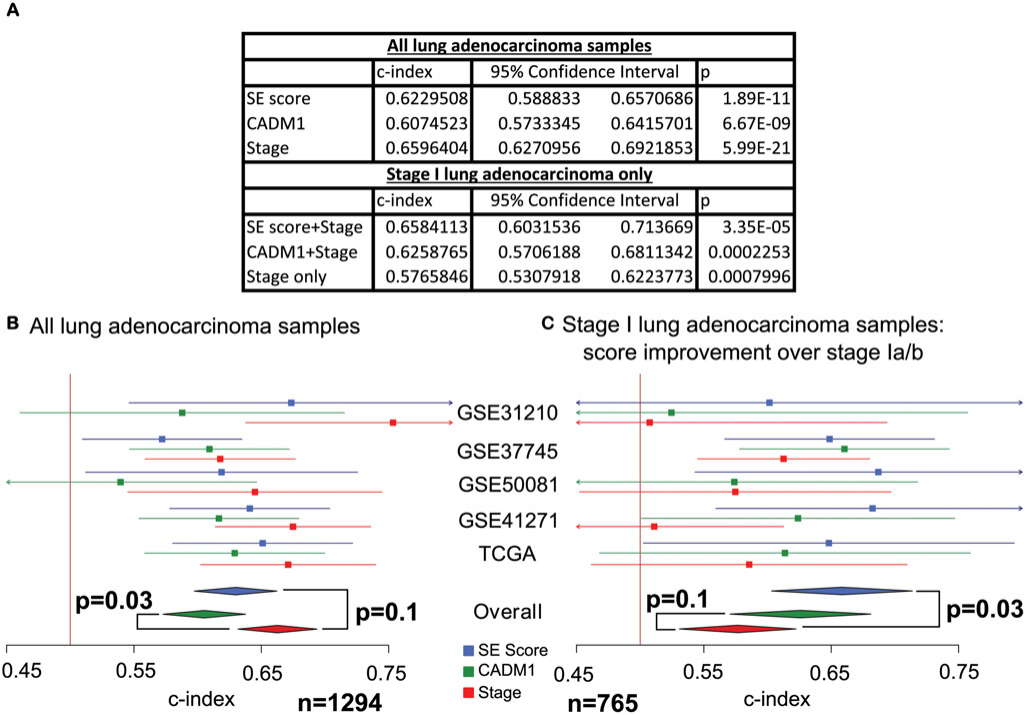
Meta-analysis comparison of the lung cancer SE score with the expression of *CADM1*. A) Survival analysis statistics for the SE score and *CADM1* expression over all samples and *stage I* samples, statistics across *stage I* samples are provided for the 2 scores combined with *stage Ia/b* status, c-index denotes concordance index and p denotes *p*-value. B) The plots display the concordance index for the SE score, *CADM1* expression and pathological tumour stage in each data set alongside 95% confidence intervals. The overall concordance indices were derived and compared via meta-analysis using a random effects model. The vertical line denotes concordance index = 0.5, data sets where the confidence interval for the concordance index crosses this line did not reach significance. Meta-analysis across 5 validation data sets reveals that the SE score performs comparably to tumour stage, whilst *CADM1* expression is outperformed by tumour stage. (C) The plots display the concordance index for the SE score and *CADM1* expression combined with *stage Ia/b* status, as well as *stage Ia/b* status alone, for *stage I* samples in each data set alongside 95% confidence intervals. Meta-analysis across 5 validation data sets reveals that only the SE score adds prognostic value to *stage Ia/b* status.

Meta-analysis across 5 independent validation data sets revealed that the SE score is prognostic across all samples and across *stage I* samples (all samples: c-index = 0.62, 95% CI = (0.59, 0.66), *p* = 1.9*e* − 11, *stage I*: c-index = 0.66, 95% CI = (0.60, 0.71), *p* = 3.35*e* − 5, Fig. 5A).

We next compared our SE score to a leading gene expression based prognostic indicator for lung adenocarcinoma, the expression of the gene *CADM1*, which was recently found to be a superior prognostic indicator to many others in the literature [44]. *CADM1* expression performed comparably to the SE score in a meta-analysis, however, it was outperformed by pathological tumour stage (*CADM1* expression vs stage: *p* = 0.03). In contrast the SE score performed comparably to tumour stage (SE score vs stage: *p* = 0.13, Fig. 5B).

Conventional tumour sub staging by size within the *stage I* stratum, is established clinical practice, it has thus been suggested that prognostic scores should aim to provide information which complements this staging, rather than seeks to replace it [49]. We therefore evaluated whether prognostic models which combined either the SE score or *CADM1* expression with *stage Ia/b* status within the *stage I* sub group, outperformed *stage Ia/b* status alone. We found that the SE score improved over *stage Ia/b* alone in a meta-analysis across 765 *stage I* lung adenocarcinomas (SE score+stage vs stage: *p* = 0.025), whereas *CADM1* expression made no improvement over *stage Ia/b* (*CADM1* expression+stage vs stage: *p* = 0.13, Fig. 5C). Whence it may be argued that the SE score provides a stronger candidate prognostic tool than *CADM1* expression for clinical application.

Another popular prognostic score for lung adenocarcinoma was derived recently by Kratz *et al*. [22], similarly to OncotypeDX however, this score is based on RT-PCR and thus a direct comparison is difficult. However, a microarray based approximation of the Kratz *et al*. score was found to perform comparably to signalling entropy both across all samples (SE score vs Kratz *et al.* score: *p* = 0.21) and across *stage I* samples (SE score vs Kratz *et al.* score: *p* = 0.37, S7 Fig).

### The prognostic impact of signalling entropy is associated with genes involved in cancer stem cells and treatment resistance

Given the power of signalling entropy as a prognostic factor in both breast and lung cancer we next investigated which genes and pathways were associated with signalling entropy’s prognostic impact, independently of other clinical variables.

To determine which gene sets were enriched among the genes prognostically related to signalling entropy independently of other variables, we considered for breast cancer a list of 320 genes which were prognostic, independent of ER status and grade, and correlated with signalling entropy, again independently of ER status and grade, in both MEATBRIC datasets. For lung adenocarcinoma we considered a list of 158 genes identified as prognostic independently of stage, and correlated with signalling entropy, again independently of stage, in both the Director’s Challenge and TCGA data sets. The two gene lists displayed an overlap of 47 genes (S5 Table displays both gene lists). We performed a gene set enrichment analysis, using a Fisher’s Exact test, comparing each of these gene lists separately against the Molecular Signatures Database [50] (S6 Table shows the top 10 enriched gene sets for both gene lists). The decision to use these gene sets for the enrichment screens, rather than the genes utilised to derive the SE scores was due to them being derived from multiple data sets and thus more robustly representative of signalling entropy’s prognostic associations. We note that gene set enrichment analysis performed on the genes comprising the SE scores gave broadly similar results (S7 Table).

The genes found to prognostically associate with signalling entropy in both lung and breast cancer showed considerable concordance in enrichment profiles (even after removal of the 47 genes in the overlap S6 Table). The strongest enrichment was for genes associated with poor survival in lung cancer, histological grade in breast cancer and cell proliferation, supporting the notion that signalling entropy is a prognostic measure of cell anaplasia. In addition, considerable enrichment was found for genes down regulated by the therapeutic agent salirasib and by *EGFR* inhibitors, as well as for genes as up regulated in cell lines resistant to the chemotherapeutic doxorubicin, supporting the hypothesis that signalling entropy associates with therapeutic resistance.

Enrichment was also found for gene sets associated with stem cells and certain CSC pathways. Examples include, genes down-regulated by *EZH2*, a well known stem cell gene involved in the pathogenesis of several cancers and which plays a documented role in both breast and lung CSCs [51–54]. The set of genes down regulated by *CTNNB1* knock-out, a critical component of the Wnt signalling pathway, posited to be important in CSCs and their therapeutic resistance [55] were also enriched. Targets of *BMP2* were among the most enriched gene sets in breast but not lung cancer, which is intriguing given the role of this gene specifically in breast CSCs [56]. Enrichment was also found for many gene sets associated with immune system processes.

Thus signalling entropy is prognostically related to genes associated with both CSCs and treatment resistance, across multiple malignancies and independently of clinical variables. This result confirms our initial postulate that signalling entropy is a powerful prognostic measure, related both to cell anaplasia and CSCs as well as treatment resistance.

## Discussion

The discovery that CSCs show resistance to conventional therapy necessitates an evaluation of their prognostic and predictive value, as well as the development of targeted therapies [8, 9]. The notion of tumour cell plasticity raises further challenges [57] with recent discoveries suggesting that CSCs may arise from the tumour bulk by simple changes [58]. This calls into question the notion that CSCs only ever occupy a small proportion of the tumour, and paint a picture of cancer cells as malleable entities capable of generating considerable heterogeneity. Recent observations have also demonstrated the importance of characterising such intra-tumour heterogeneity in the prognostic assessment of epithelial cancers [17]. The measurement of both CSC abundance and intra-tumour heterogeneity in a clinically relevant manner, however, presents a challenge [59]. The majority of currently suggested approaches are limited in sample size, and require the time consuming collection of large new data sets (such as multiple biopsies from single tumours) for validation and proof of concept.

Here we have shown that signalling entropy, a measure of pathway promiscuity, which is elevated in CSCs as compared to the tumour bulk is also a potential correlate of intra-tumour heterogeneity. Importantly, our measure is applicable to the plethora of publicly available bulk tumour, genome wide expression data, facilitating swift validation of its prognostic impact on large data sets. By considering 5360 primary tumour samples, we have demonstrated that our measure is a powerful prognostic indicator in both breast and lung cancer. In breast cancer our measure is prognostic within the grade 2 stratum and both ER positive and negative subtypes. In lung adenocarcinoma, our measure is prognostic within the *stage I* stratum, out-performing tumour size.

Signalling entropy is computed from the expression of many thousands of genes and thus is not swiftly translatable. Moreover, it is associated with yet prognostically independent of a number of clinical variables in both breast and lung cancer. We thus used feature selection to derive a small set of genes which capture the prognostic power of signalling entropy independently of other clinical variables, thus representing a more readily applicable quantifier of stemness and intra-tumour heterogeneity.

Expression based prognostic indicators for epithelial cancers have been a topic of considerable interest in recent years [22–25, 44, 60]. Arguably the most successful application has been to breast cancer, where OncotypeDX and MammaPrint are currently in clinical trials for guiding the management of ER positive breast cancer [26, 27]. Though powerful, these assays are limited to the ER positive subtype and importantly ignore CSC abundance and intra-tumour heterogeneity. There also exist many more sophisticated prognostic signatures for breast cancer, derived from within the DREAM challenge consortium, and several of which have demonstrated improvement over MammaPrint or OncotypeDX [61–64]. The aim of our work, was first to introduce a prognostic measure of signalling promiscuity, which by approximating CSC abundance and intra-tumour heterogeneity may prove a basis by which to improve the construction of prognostic models for epithelial cancers, and secondly, to compare it to clinically well established or validated signatures such as MammaPrint and OncotypeDX. A direct comparison of signalling entropy to the prognostic indicators from the DREAM challenge, which have not yet entered the clinical setting, is beyond the scope of this work.

In comparing signalling entropy to signatures such as MammaPrint it is worth pointing out that a direct comparison is unfair signalling entropy does not involve feature selection. Even so, signalling entropy was found to be more robust than MammaPrint across ER+ and ER- breast cancer. Although signalling entropy was not found to outperform existing prognostic markers in lung adenocarcinoma, by using the SE score, derived by signalling entropy guided feature selection, it was possible to outperform existing state of the art prognostic factors such as *CADM1* expression across independent data sets.

The nature of signalling entropy as a measure of pathway promiscuity, which correlates with CSCs and associates with intra-tumour heterogeneity [8, 9], led us to postulate that it may associate with the phenotypic plasticity of a tumour that enables subversion of therapeutic response. Here we demonstrated that signalling entropy’s prognostic power in epithelial cancers is indeed related to both treatment resistance and CSC pathways.

We thus propose signalling entropy as a powerful and readily applicable tool for assessing the prognostic impact of signalling promiscuity across multiple epithelial cancers. In addition to being a strong prognostic factor which outperforms the leading expression based indicators, our measure may also provide insights into intra-tumour heterogeneity, treatment resistance and CSC mechanisms.

## Materials and Methods

Details of data sets used, the interaction network and all statistical methods can be found in the S1 Text.

### Signalling Entropy

Signalling entropy was computed in a sample specific manner as described in [16]. Briefly, each sample is first integrated with a Protein Interaction Network (PIN) (see S1 Text) to create a sample specific stochastic matrix, *P* = (*p*_*ij*_). By integrating each sample with the PIN, rather than considering a complete network in which every protein pair can directly interact, we benefit both from a reduction in computational complexity and an improved biological relevance from a focus on direct interactions. Integration with the PIN filters out indirect interactions even if strong correlations are present, making our analysis robust to confounding effects. By using each sample to weigh the PIN we are also reducing the noise present in the network by providing it with a sample-specific biological context. The *i* ^*th*^ row of *P* defines a probability distribution describing the rates of reaction of protein *i* with each of its neighbours in the PIN. These distributions are constructed by appealing to a simplified version of the mass action principle, namely that the rate of a reaction is proportional to the product of the active masses of the reagents involved. We assume that log normalised gene expression is a rough proxy for protein concentration and thus compute *P* as follows:

$$\begin{array}{c} p_{ij}=\left\{\begin{array}{cc} E_{j}/\sum_{k\in N(i)}E_{k}, & \text{if}\;j\in N(i)\\ 0, & \text{else} \end{array}\right. \end{array}$$ (1)

where *E*_*j*_ is the log-normalised expression of gene *j* in the given sample and *N*(*i*) denotes the set of direct interaction partners (neighbours) of gene *i* in the PIN. We note that from this definition ∑_*j*_ *p*_*ij*_ = 1 for all *j*, *i.e*., *P* is row stochastic, and the *i* ^*th*^ row corresponds to the weighted interaction distribution of protein *i* in the given sample. We note that not all proteins in the PIN have a corresponding probe in the microarray or sequence in the RNA-seq data, consequentially the PIN we consider is the maximally connected component of the original PIN after the removal of missing proteins.

For each protein *i* we then define the local entropy of its interaction distribution, *S*_*i*_, which quantifies the promiscuity of its signalling within the sample:

$$\begin{array}{c} S_{i}=-\sum_{j\in N(i)}p_{ij}\log p_{ij}. \end{array}$$ (2)

Signalling entropy is a global measure of signalling promiscuity in a given sample and thus is computed from the entire stochastic matrix *p*_*ij*_ as the entropy rate, *S̃*_*R*_, of the stochastic process described by *p*_*ij*_:

$$\begin{array}{c} {\tilde{S}}_{R}=\sum_{i}\pi_{i}S_{i}, \end{array}$$ (3)

where *π*_*i*_ denotes the stationary distribution of the stochastic matrix, satisfying ∑_*i*_ *π*_*i*_ *p*_*ij*_ = *π*_*j*_. We note that *π*_*i*_ is therefore the non-degenerate eigenvector of *P* corresponding to the eigenvalue 1 and that by the Perron Frobenius theorem, the existence of *π*_*i*_ requires that the matrix *P* be irreducible; this is guaranteed by the fact that the PIN considered is connected and non-bipartite [65].

The maximum entropy rate of a weighted network, *M*_*R*_, depends solely upon its adjacency matrix, *A* = (*A*_*ij*_), and can be calculated as the entropy rate of the stochastic matrix *p*_*ij*_ = *A*_*ij*_ *ν*_*j*_/*λν*_*i*_, where *λ* and *ν* are the dominant eigenvalue and corresponding eigenvector of *A*, respectively [66]. In order to ensure the results presented in this paper are comparable with those of previous studies on signalling entropy, we will present our findings in terms of normalised signalling entropy:

$$\begin{array}{c} S_{R}={\tilde{S}}_{R}/M_{R}. \end{array}$$ (4)

A closed form expression for signalling entropy is derived and analysed in the S1 Text. R-scripts for the computation of signalling entropy are freely available for download at www.sourceforge.net/projects/signalentropy.

### Super-additivity and heterogeneity

We hypothesised that the signalling entropy of a heterogeneous sample generated from a 50:50 mixture of two homogeneous cell types will be greater, on average, than the signalling entropy of a homogeneous sample. Here we show that if signalling entropy is super-additive then the hypothesis is correct. Let us first define some preliminaries: Let *x*_*i*_ ∈ ℝ^> 0^ be the expression of gene *i* in cell type *X*, and denote the vector containing all such variables by $x={\left(x_{i}\right)}_{i=1}^{n}\in \Omega \subset {\mathbb{R}}^{>0}$, where Ω is some bounded domain. In our analysis *x* will represent the vector of log normalised gene expression values for a homogeneous sample, we note that as the expression of genes cannot be infinite we bound *x* within a finite domain Ω, of biologically admissible expression regimes.

Our hypothesis on signalling entropy thus amounts to proving the following proposition:

**Proposition**. *Let x, y* ∈ Ω, *then*

$$\begin{array}{c} \underset{\Omega }{\int^{\text{​}}}\underset{\Omega }{\int^{\text{​}}}\left(S_{R}\left(\frac{x+y}{2}\right)-S_{R}\left(x\right)\right)dxdy>0. \end{array}$$ (5)

Let us consider a the following claim:

**Claim** **(Super-additivity)**. *Let x, y* ∈ Ω *then*

$$\begin{array}{c} S_{R}\;\left(\frac{x+y}{2}\right)>\frac{S_{R}\left(x\right)}{2}+\frac{S_{R}\left(y\right)}{2}. \end{array}$$ (6)

It is clear that if the claim is true then the proposition must be true. Notice first that if the claim is true then as it is a strict bound ∃*ε* > 0 such that $S_{R}\left(\frac{x+y}{2}\right)>\frac{S_{R}(x)}{2}+\frac{S_{R}(y)}{2}+\varepsilon$. Whence

$$\begin{array}{ccc} \int_{\Omega }\int_{\Omega }\left(S_{R}\;\left(\frac{x+y}{2}\right)-S_{R}\;\left(x\right)\right)\;dxdy & > & \int_{\Omega }\int_{\Omega }\left(\frac{S_{R}\left(y\right)}{2}-\frac{S_{R}\left(x\right)}{2}+\epsilon \right)\;dxdy \end{array}$$ (7)

$$\begin{array}{ccc} & = & {\left|\Omega \right|}^{2}\epsilon \end{array}$$ (8)

$$\begin{array}{ccc} & > & 0, \end{array}$$ (9)

and thus the proposition is true.

Thus if signalling entropy is super-additive over homogeneous cell types, this implies that signalling entropy will on average be elevated in heterogeneous mixtures of cell types. These propositions are examined in detail in S1 Text.

## Supporting Information

S1 Text This document contains supplementary materials and methods and supplementary results to complement the manuscript. (PDF)

S1 Table GEO and ArrayExpress accession numbers for the breast cancer and lung adenocarcinoma data sets. Sample counts are provided for ER segregated and grade 2 samples in the case of breast cancer, and also for *stage I* samples in the case of lung adenocarcinoma.
(XLSX)

S2 Table The genes utilised to construct the signalling entropy prognostic score in breast cancer, derived from the METABRIC discovery set. Genes are separated into those found to positively correlate with signalling entropy and those negatively correlated.
(XLSX)

S3 Table The genes utilised to construct the signalling entropy prognostic score derived from the METABRIC validation set. Genes are separated into those found to positively correlate with signalling entropy and those negatively correlated, the genes which overlap with the discovery set derived set are highlighted in yellow. Also presented are genes which are prognostic independently of ER status and grade in both discovery and validation sets of METABRIC (middle table). Genes which are both prognostic and correlated with signalling entropy, independently of ER status and grade, in both discovery and validation sets of METABRIC are presented as the rightmost table.
(XLSX)

S4 Table The genes utilised to construct the signalling entropy prognostic score in lung adenocarcinoma. Genes are separated into those found to positively correlate with signalling entropy and those negatively correlated.
(XLSX)

S5 Table Genes utilised in the gene set enrichment analysis to identify gene sets associated with signalling entropy’s prognostic power in breast and lung cancer. In the case of breast cancer these are prognostic genes which correlate with signalling independently of ER status and grade and whose prognostic power is also independent of these variables, in both METABRIC data sets. In the case of lung cancer, these are prognostic genes which are correlated with signalling entropy independently of tumour stage and whose prognostic power is also independent of stage, in both the TCGA and Director’s Challenge lung adenocarcinoma data sets. Genes are separated into those found to positively correlate with signalling entropy and those negatively correlated.
(XLSX)

S6 Table Gene set enrichment analysis results displaying the top 10 most significant enriched gene sets associated with signalling entropy’s prognostic power in breast and lung cancer. Tables display results for the gene set enrichment analysis performed on gene lists identified in lung and breast cancer separately, both with and without the intersection of the two lists removed.
(XLSX)

S7 Table Gene set enrichment analysis results displaying the top 10 most significant enriched gene sets associated with the genes utilised to construct the SE score in both breast cancer and lung adenocarcinoma. (XLSX)

S1 Fig Signalling entropy is correlated with both the Ben-Porath *et al*. and Sotiriou *et al*. tumour grade signatures. The *p*-values denote the significance of the Pearson correlation coefficient.
(EPS)

S2 Fig Signalling entropy outperforms the Ben-Porath ES cell signature in measuring tumour grade. A) Signalling entropy is associated with histological tumour grade. B) Unlike signalling entropy the Ben-Porath *et al*. signature cannot discriminate between grade 1 and grade 2 breast cancers in the METABRIC discovery data set. All *p*-values are derived from Wilcoxon tests.
(EPS)

S3 Fig Prognostic associations of random gene expression signatures in METABRIC. A) Kaplan-Meyer plots for 5 year censored survival data are presented for each of the 3 random gene expression signatures described by Venet *et al.* in each METABRIC data set, *p*-values denote the significance of a Cox-regression for each random signature as assessed by a Wald-test. We see that only KRISHNAN2007DEFEAT is significantly prognostic in both METABRIC datasets. B) Kaplan-Meyer plots for 5 year censored survival data are presented for each of the KRISHNAN2007DEFEAT expression signature in each METABRIC data set, divided into ER+ and ER- samples, *p* values denote the significance of a Cox-regression for each random signature as assessed by a Wald-test. We see that the random signature is not prognostic within ER subtypes.
(EPS)

S4 Fig Meta-analysis comparison of signalling entropy with OncotypeDX. The plots display the concordance indices for signalling entropy and a microarray based approximation of OncotypeDX in each data set alongside 95% confidence intervals. The overall concordance indices were derived via meta-analysis using a random effects model. The vertical line denotes concordance index = 0.5, data sets where the confidence interval for the concordance index crosses this line did not reach significance. Meta-analysis across 10 data sets reveals that signalling entropy performs comparably to OncotypeDX across (A) ER positive samples and (B) ER negative samples.
(EPS)

S5 Fig Signalling entropy is associated with the level of tumour differentiation in lung adenocarcinoma in the Director’s Challenge dataset. A) Signalling entropy is correlated with the Ben-Porath *et al*. tumour grade signature, the *p*-value denotes the significance of the Pearson correlation coefficient. B) Signalling entropy is associated with histological tumour grade, *p*-values are derived from Wilcoxon tests.
(EPS)

S6 Fig Signalling entropy is elevated in lung adenocarcinoma patients with a history of smoking. *p*-values are derived from Wilcoxon tests.
(EPS)

S7 Fig Meta-analysis comparison of signalling entropy with the score of Kratz *et al*. A) The plots display the concordance indices for signalling entropy and a microarray based approximation of the Kratz *et al*. score in each data set alongside 95% confidence intervals. The overall concordance indices were derived via meta-analysis using a random effects model. The vertical line denotes concordance index = 0.5, data sets where the confidence interval for the concordance index crosses this line did not reach significance. Meta-analysis across 6 data sets reveals that signalling entropy performs comparably to the Kratz *et al*. score across all samples. (B) The plots display the concordance indices for signalling entropy and the Kratz *et al*. score combined with *stage Ia/b* status for *stage I* samples in each data set alongside its 95% confidence interval. Meta-analysis across 6 data sets reveals that signalling entropy performs comparably to the score of Kratz *et al*..
(EPS)

S8 Fig Analysis of the expression *sign*(1 − 1/*b* + 2/*a*) + *sign*(1 − *a* + 2*b*), which evaluates to 2 if signalling entropy is super-additive and is derived in the S1 Text, for a range of biologically plausible values of *a* and *b*, parameters derived in the S1 Text. A) Histogram of values of the *sign*(1 − 1/*b* + 2/*a*) + *sign*(1 − *a* + 2*b*) evaluated over 2000 equally incremented values of *a* and *b* over the range *a*, *b* ∈ [0.01, 20].
We see that the majority of the values satisfy the condition *sign*(1 − 1/*b* + 2/*a*) + *sign*(1 − *a* + 2*b*) = 2. B) Plot of *sign*(1 − 1/*b* + 2/*a*) + *sign*(1 − *a* + 2*b*) for *a*, *b* ∈ [0.01, 20], values of *a* are plotted on the *x* axis whilst colors from red to green to blue denote increasing values of *b*, we see that as *a* and *b* increase the expression quickly evaluates to 2.
(EPS)

S9 Fig Demonstration that the claim $S_{R}\;\left(\frac{x+y}{2}\right)>\frac{S_{R}(x)}{2}+\frac{S_{R}(y)}{2}$ is correct for all pairwise combinations of samples in GSE2361. (EPS)

S10 Fig Signalling entropy of homogeneous and mixed tissues. The first box represents the signalling entropy distribution of 33 unmixed tissues, whilst each subsequent labelled box represents the signalling entropy distribution of the labelled tissue mixed with each of the remaining 32 tissues. The red line represents the median of the unmixed samples. We see that for 20/33 tissue types, the median of the mixture is greater than the median of the pure samples, suggesting that on average the signalling entropy of the mixture is greater than the signalling entropy of the pure sample.
(EPS)

S11 Fig Demonstration that the claim, $\int_{\Omega }\int_{\Omega }\left(S_{R}\;\left(\frac{x+y}{2}\right)-S_{R}(x)\right)\;dxdy>0$ is correct for samples in GSE2361. The *p*-value is for a paired Wilcoxon test.
(EPS)

## References

[1] Reya T, Morrison SJ, Clarke MF, Weissman IL (2001) Stem cells, cancer, and cancer stem cells. Nature 414: 105–11. 10.1038/35102167 11689955

[2] Stingl J, Caldas C (2007) Molecular heterogeneity of breast carcinomas and the cancer stem cell hypothesis. Nat Rev Cancer 7: 791–9. 10.1038/nrc2212 17851544

[3] Shackleton M, Quintana E, Fearon ER, Morrison SJ (2009) Heterogeneity in cancer: cancer stem cells versus clonal evolution. Cell 138: 822–9. 10.1016/j.cell.2009.08.017 19737509

[4] Nowell PC (1976) The clonal evolution of tumor cell populations. Science 194: 23–8. 10.1126/science.959840 959840

[5] Heppner GH (1984) Tumor heterogeneity. Cancer Res 44: 2259–65. 6372991

[6] Fidler IJ, Hart IR (1982) Biological diversity in metastatic neoplasms: origins and implications. Science 217: 998–1003. 10.1126/science.7112116 7112116

[7] Al-Hajj M, Wicha MS, Benito-Hernandez A, Morrison SJ, Clarke MF (2003) Prospective identification of tumorigenic breast cancer cells. Proc Natl Acad Sci U S A 100: 3983–8. 10.1073/pnas.0530291100 12629218 PMC153034

[8] Pinto CA, Widodo E, Waltham M, Thompson EW (2013) Breast cancer stem cells and epithelial mesenchymal plasticity—implications for chemoresistance. Cancer Lett 341: 56–62. 10.1016/j.canlet.2013.06.003 23830804

[9] Creighton CJ, Li X, Landis M, Dixon JM, Neumeister VM, Sjolund A, et al (2009) Residual breast cancers after conventional therapy display mesenchymal as well as tumor-initiating features. Proc Natl Acad Sci U S A 106: 13820–5. 10.1073/pnas.0905718106 19666588 PMC2720409

[10] de Beca FF, Caetano P, Gerhard R, Alvarenga CA, Gomes M, Paredes J, et al (2013) Cancer stem cells markers CD44, CD24 and ALDH1 in breast cancer special histological types. J Clin Pathol 66: 187–91. 10.1136/jclinpath-2012-201169 23112116

[11] Bruna A, Greenwood W, Le Quesne J, Teschendorff A, Miranda-Saavedra D, Rueda OM, et al (2012) TGF-beta induces the formation of tumour-initiating cells in claudinlow breast cancer. Nat Commun 3: 1055 10.1038/ncomms2039 22968701

[12] Ben-Porath I, Thomson MW, Carey VJ, Ge R, Bell GW, Regev A, et al (2008) An embryonic stem cell-like gene expression signature in poorly differentiated aggressive human tumors. Nat Genet 40: 499–507. 10.1038/ng.127 18443585 PMC2912221

[13] Hassan KA, Chen G, Kalemkerian GP, Wicha MS, Beer DG (2009) An embryonic stem cell-like signature identifies poorly differentiated lung adenocarcinoma but not squamous cell carcinoma. Clin Cancer Res 15: 6386–90. 10.1158/1078-0432.CCR-09-1105 19808871 PMC2787085

[14] Prat A, Parker JS, Karginova O, Fan C, Livasy C, Herschkowitz JI, et al (2010) Phenotypic and molecular characterization of the claudin-low intrinsic subtype of breast cancer. Breast Cancer Res 12: R68 10.1186/bcr2635 20813035 PMC3096954

[15] Taube JH, Herschkowitz JI, Komurov K, Zhou AY, Gupta S, Yang J, et al (2010) Core epithelial-to-mesenchymal transition interactome gene-expression signature is associated with claudin-low and metaplastic breast cancer subtypes. Proc Natl Acad Sci U S A 107: 15449–54. 10.1073/pnas.1004900107 20713713 PMC2932589

[16] Banerji CRS, Miranda-Saavedra D, Severini S, Widschwendter M, Enver T, Zhou JX, et al (2013) Cellular network entropy as the energy potential in waddington’s differentiation landscape. Sci Rep 3: 3039 10.1038/srep03039 24154593 PMC3807110

[17] Gerlinger M, Rowan AJ, Horswell S, Larkin J, Endesfelder D, Gronroos E, et al (2012) Intratumor heterogeneity and branched evolution revealed by multiregion sequencing. N Engl J Med 366: 883–92. 10.1056/NEJMoa1113205 22397650 PMC4878653

[18] Maley CC, Galipeau PC, Finley JC, Wongsurawat VJ, Li X, Sanchez CA, et al (2006) Genetic clonal diversity predicts progression to esophageal adenocarcinoma. Nat Genet 38: 468–73. 10.1038/ng1768 16565718

[19] Vogelstein B, Papadopoulos N, Velculescu VE, Zhou S, Diaz J L A, Kinzler KW (2013) Cancer genome landscapes. Science 339: 1546–58. 10.1126/science.1235122 23539594 PMC3749880

[20] West J, Bianconi G, Severini S, Teschendorff AE (2012) Differential network entropy reveals cancer system hallmarks. Sci Rep 2: 802 10.1038/srep00802 23150773 PMC3496163

[21] Siegel R, Naishadham D, Jemal A (2013) Cancer statistics, 2013. CA Cancer J Clin 63: 11–30. 10.3322/caac.21166 23335087

[22] Kratz JR, He J, Van Den Eeden SK, Zhu ZH, Gao W, Pham PT, et al (2012) A practical molecular assay to predict survival in resected non-squamous, non-small-cell lung cancer: development and international validation studies. Lancet 379: 823–32. 10.1016/S0140-6736(11)61941-7 22285053 PMC3294002

[23] Bergot E, Levallet G, Campbell K, Dubois F, Lechapt E, Zalcman G (2013) Predictive biomarkers in patients with resected non-small cell lung cancer treated with perioperative chemotherapy. Eur Respir Rev 22: 565–76. 10.1183/09059180.00007113 24293473 PMC9639177

[24] van’t Veer LJ, Dai H, van de Vijver MJ, He YD, Hart AA, Mao M, et al (2002) Gene expression profiling predicts clinical outcome of breast cancer. Nature 415: 530–6. 10.1038/415530a 11823860

[25] van de Vijver MJ, He YD, van’t Veer LJ, Dai H, Hart AA, Voskuil DW, et al (2002) A gene-expression signature as a predictor of survival in breast cancer. N Engl J Med 347: 1999–2009. 10.1056/NEJMoa021967 12490681

[26] Andre F, Delaloge S (2010) First-generation genomic tests for breast cancer treatment. Lancet Oncol 11: 6–7. 10.1016/S1470-2045(09)70347-X 20005177

[27] Cardoso F, Piccart-Gebhart M, Van’t Veer L, Rutgers E (2007) The mindact trial: the first prospective clinical validation of a genomic tool. Mol Oncol 1: 246–51. 10.1016/j.molonc.2007.10.004 19383299 PMC5543876

[28] van Wieringen WN, van der Vaart AW (2011) Statistical analysis of the cancer cell’s molecular entropy using high-throughput data. Bioinformatics 27: 556–63. 10.1093/bioinformatics/btq704 21172912

[29] Ge X, Yamamoto S, Tsutsumi S, Midorikawa Y, Ihara S, Wang SM, et al (2005) Interpreting expression profiles of cancers by genome-wide survey of breadth of expression in normal tissues. Genomics 86: 127–41. 10.1016/j.ygeno.2005.04.008 15950434

[30] Curtis C, Shah SP, Chin SF, Turashvili G, Rueda OM, Dunning MJ, et al (2012) The genomic and transcriptomic architecture of 2,000 breast tumours reveals novel subgroups. Nature 486: 346–52. 10.1038/nature10983 22522925 PMC3440846

[31] Sotiriou C, Wirapati P, Loi S, Harris A, Fox S, Smeds J, et al (2006) Gene expression profiling in breast cancer: understanding the molecular basis of histologic grade to improve prognosis. J Natl Cancer Inst 98: 262–72. 10.1093/jnci/djj052 16478745

[32] Venet D, Dumont JE, Detours V (2011) Most random gene expression signatures are significantly associated with breast cancer outcome. PLoS Comput Biol 7: e1002240 10.1371/journal.pcbi.1002240 22028643 PMC3197658

[33] Miller LD, Smeds J, George J, Vega VB, Vergara L, Ploner A, et al (2005) An expression signature for p53 status in human breast cancer predicts mutation status, transcriptional effects, and patient survival. Proc Natl Acad Sci U S A 102: 13550–5. 10.1073/pnas.0506230102 16141321 PMC1197273

[34] Pawitan Y, Bjohle J, Amler L, Borg AL, Egyhazi S, Hall P, et al (2005) Gene expression profiling spares early breast cancer patients from adjuvant therapy: derived and validated in two population-based cohorts. Breast Cancer Res 7: R953–64. 10.1186/bcr1325 16280042 PMC1410752

[35] Desmedt C, Piette F, Loi S, Wang Y, Lallemand F, Haibe-Kains B, et al (2007) Strong time dependence of the 76-gene prognostic signature for node-negative breast cancer patients in the transbig multicenter independent validation series. Clin Cancer Res 13: 3207–14. 10.1158/1078-0432.CCR-06-2765 17545524

[36] Kao KJ, Chang KM, Hsu HC, Huang AT (2011) Correlation of microarray-based breast cancer molecular subtypes and clinical outcomes: implications for treatment optimization. BMC Cancer 11: 143 10.1186/1471-2407-11-143 21501481 PMC3094326

[37] Loi S, Haibe-Kains B, Desmedt C, Lallemand F, Tutt AM, Gillet C, et al (2007) Definition of clinically distinct molecular subtypes in estrogen receptor-positive breast carcinomas through genomic grade. J Clin Oncol 25: 1239–46. 10.1200/JCO.2006.07.1522 17401012

[38] Wang Y, Klijn JG, Zhang Y, Sieuwerts AM, Look MP, Yang F, et al (2005) Gene-expression profiles to predict distant metastasis of lymph-node-negative primary breast cancer. Lancet 365: 671–9. 10.1016/S0140-6736(05)70933-8 15721472

[39] Chin K, DeVries S, Fridlyand J, Spellman PT, Roydasgupta R, Kuo WL, et al (2006) Genomic and transcriptional aberrations linked to breast cancer pathophysiologies. Cancer Cell 10: 529–41. 10.1016/j.ccr.2006.10.009 17157792

[40] Schmidt M, Bohm D, von Torne C, Steiner E, Puhl A, Pilch H, et al (2008) The humoral immune system has a key prognostic impact in node-negative breast cancer. Cancer Res 68: 5405–13. 10.1158/0008-5472.CAN-07-5206 18593943

[41] Viale G, Slaets L, Bogaerts J, Rutgers E, van’t Veer L, Piccart-Gebhart MJ, et al (2014) High concordance of protein (by ihc), gene (by fish; her2 only), and microarray readout (by targetprint) of er, pgr, and her2: results from the eortc 10041/big 03–04 mindact trial. Ann Oncol 25: 816–23. 10.1093/annonc/mdu026 24667714 PMC3969556

[42] Shedden K, Taylor JM, Enkemann SA, Tsao MS, Yeatman TJ, Gerald WL, et al (2008) Gene expression-based survival prediction in lung adenocarcinoma: a multi-site, blinded validation study. Nat Med 14: 822–7. 10.1038/nm.1790 18641660 PMC2667337

[43] Yamauchi M, Yamaguchi R, Nakata A, Kohno T, Nagasaki M, Shimamura T, et al (2012) Epidermal growth factor receptor tyrosine kinase defines critical prognostic genes of stage i lung adenocarcinoma. PLoS One 7: e43923 10.1371/journal.pone.0043923 23028479 PMC3446964

[44] Botling J, Edlund K, Lohr M, Hellwig B, Holmberg L, Lambe M, et al (2013) Biomarker discovery in non-small cell lung cancer: integrating gene expression profiling, meta-analysis, and tissue microarray validation. Clin Cancer Res 19: 194–204. 10.1158/1078-0432.CCR-12-1139 23032747

[45] Der SD, Sykes J, Pintilie M, Zhu CQ, Strumpf D, Liu N, et al (2014) Validation of a histology-independent prognostic gene signature for early-stage, non-small-cell lung cancer including stage ia patients. J Thorac Oncol 9: 59–64. 24305008 10.1097/JTO.0000000000000042

[46] Sato M, Larsen JE, Lee W, Sun H, Shames DS, Dalvi MP, et al (2013) Human lung epithelial cells progressed to malignancy through specific oncogenic manipulations. Mol Cancer Res 11: 638–50. 10.1158/1541-7786.MCR-12-0634-T 23449933 PMC3687022

[47] Ein-Dor L, Kela I, Getz G, Givol D, Domany E (2005) Outcome signature genes in breast cancer: is there a unique set? Bioinformatics 21: 171–8. 10.1093/bioinformatics/bth469 15308542

[48] Michiels S, Koscielny S, Hill C (2005) Prediction of cancer outcome with microarrays: a multiple random validation strategy. Lancet 365: 488–92. 10.1016/S0140-6736(05)17866-0 15705458

[49] Subramanian J, Simon R (2010) Gene expression-based prognostic signatures in lung cancer: ready for clinical use? J Natl Cancer Inst 102: 464–74. 10.1093/jnci/djq025 20233996 PMC2902824

[50] Liberzon A, Subramanian A, Pinchback R, Thorvaldsdottir H, Tamayo P, Mesirov JP (2011) Molecular signatures database (MSigDB) 3.0. Bioinformatics 27: 1739–40. 10.1093/bioinformatics/btr260 21546393 PMC3106198

[51] Kleer CG, Cao Q, Varambally S, Shen R, Ota I, Tomlins SA, et al (2003) EZH2 is a marker of aggressive breast cancer and promotes neoplastic transformation of breast epithelial cells. Proc Natl Acad Sci U S A 100: 11606–11. 10.1073/pnas.1933744100 14500907 PMC208805

[52] Gonzalez ME, Moore HM, Li X, Toy KA, Huang W, Sabel MS, et al (2014) EZH2 expands breast stem cells through activation of NOTCH1 signaling. Proc Natl Acad Sci U S A 111: 3098–103. 10.1073/pnas.1308953111 24516139 PMC3939892

[53] Takawa M, Masuda K, Kunizaki M, Daigo Y, Takagi K, Iwai Y, et al (2011) Validation of the histone methyltransferase ezh2 as a therapeutic target for various types of human cancer and as a prognostic marker. Cancer Sci 102: 1298–305. 10.1111/j.1349-7006.2011.01958.x 21539681 PMC11159278

[54] Shao C, Sullivan JP, Girard L, Augustyn A, Yenerall P, Rodriguez-Canales J, et al (2014) Essential role of aldehyde dehydrogenase 1A3 for the maintenance of non-small cell lung cancer stem cells is associated with the STAT3 pathway. Clin Cancer Res 20: 4154–66. 10.1158/1078-0432.CCR-13-3292 24907115 PMC4438754

[55] Takahashi-Yanaga F, Kahn M (2010) Targeting Wnt signaling: can we safely eradicate cancer stem cells? Clin Cancer Res 16: 3153–62. 10.1158/1078-0432.CCR-09-2943 20530697

[56] Buijs JT, van der Horst G, van den Hoogen C, Cheung H, de Rooij B, Kroon J, et al (2012) The BMP2/7 heterodimer inhibits the human breast cancer stem cell subpopulation and bone metastases formation. Oncogene 31: 2164–74. 10.1038/onc.2011.400 21996751

[57] Marjanovic ND, Weinberg RA, Chaffer CL (2013) Cell plasticity and heterogeneity in cancer. Clin Chem 59: 168–79. 10.1373/clinchem.2012.184655 23220226 PMC6220421

[58] Chaffer CL, Marjanovic ND, Lee T, Bell G, Kleer CG, Reinhardt F, et al (2013) Poised chromatin at the zeb1 promoter enables breast cancer cell plasticity and enhances tumorigenicity. Cell 154: 61–74. 10.1016/j.cell.2013.06.005 23827675 PMC4015106

[59] Bedard PL, Hansen AR, Ratain MJ, Siu LL (2013) Tumour heterogeneity in the clinic. Nature 501: 355–64. 10.1038/nature12627 24048068 PMC5224525

[60] Xie Y, Minna JD (2012) A lung cancer molecular prognostic test ready for prime time. Lancet 379: 785–7. 10.1016/S0140-6736(12)60154-8 22386017 PMC3382084

[61] Bilal E, Dutkowski J, Guinney J, Jang IS, Logsdon BA, Pandey G, et al (2013) Improving breast cancer survival analysis through competition-based multidimensional modeling. PLoS Comput Biol 9: e1003047 10.1371/journal.pcbi.1003047 23671412 PMC3649990

[62] Dowsett M, Sestak I, Lopez-Knowles E, Sidhu K, Dunbier AK, Cowens JW, et al (2013) Comparison of pam50 risk of recurrence score with oncotype dx and ihc4 for predicting risk of distant recurrence after endocrine therapy. J Clin Oncol 31: 2783–90. 10.1200/JCO.2012.46.1558 23816962

[63] Cuzick J, Dowsett M, Pineda S, Wale C, Salter J, Quinn E, et al (2011) Prognostic value of a combined estrogen receptor, progesterone receptor, ki-67, and human epidermal growth factor receptor 2 immunohistochemical score and comparison with the genomic health recurrence score in early breast cancer. J Clin Oncol 29: 4273–8. 10.1200/JCO.2010.31.2835 21990413

[64] Parker JS, Mullins M, Cheang MC, Leung S, Voduc D, Vickery T, et al (2009) Supervised risk predictor of breast cancer based on intrinsic subtypes. J Clin Oncol 27: 1160–7. 10.1200/JCO.2008.18.1370 19204204 PMC2667820

[65] Grimmett G, Stirzaker D (1992) Probability and random processes. Oxford University Press.

[66] Demetrius L, Manke T (2005) Robustness and network evolution-an entropic principle. Physica A: Statistical Mechanics and its Applications 346: 682–696. 10.1016/j.physa.2004.07.011

