# Supplementary material for: Intra-Tumour Signalling Entropy Determines Clinical Outcome in Breast and Lung Cancer
Source: PLoS Comput Biol. 2015 Mar 20;11(3):e1004115. doi: 10.1371/journal.pcbi.1004115 (PMC4368751; doi:10.1371/journal.pcbi.1004115)
Supplement: S1 Text — (PDF) [file pcbi.1004115.s001.pdf]

S1 Text: Intra-tumour signalling entropy determines clinical outcome in breast and lung cancer

Christopher R. S. Banerji[1,2,3,*], Simone Severini[2], Carlos Caldas[4], Andrew E. Teschendorff[1,5]

1. Statistical Cancer Genomics, Paul O'Gorman Building, UCL Cancer Institute, University College London, London WC1E 6BT, UK.
2. Department of Computer Science, University College London, London WC1E 6BT, UK.
3. Centre of Mathematics and Physics in the Life Sciences and Experimental Biology, University College London, London WC1E 6BT, UK.
4. Breast Cancer Functional Genomics Laboratory, Cancer Research UK, Cambridge Institute, University of Cambridge, Li Ka Shing Centre, Robinson Way, Cambridge, UK.
5. CAS-MPG Partner Institute for Computational Biology, Chinese Academy of Sciences, Shanghai Institute for Biological Sciences, 320 Yue Yang Road, Shanghai 200031, China.

* Corresponding Authors: Christopher R.S. Banerji, e-mail: *christopher.banerji.11@ucl.ac.uk* and Andrew E. Teschendorff, e-mail: *a.teschendorff@ucl.ac.uk*

Supplementary Materials and Methods

Expression Data

For our initial survival studies in breast cancer and for identification of signalling entropy associations with clinical variables we considered data from 1980 primary breast cancer patients collected by the Molecular Taxonomy of Breast Cancer International Consortium (METABRIC) project [30]. The data consists of microarrays profiled on the Illumina HT 12v3 platform and log normalised as described in reference [30]. This expression data is accompanied by extensive clinical annotation describing tumour grade, size, stage, cellularity, histological subtype, lymph node status, hormone receptor status, Pfam50 subtype and p53 mutation status, as well as patient age at diagnosis, menopausal status, treatment and survival data. As described in [30] the expression data was divided into a discovery set of 997 samples and a validation set of 983 samples, each representing a distribution of clinical variables and molecular subtypes observed in the full data set.
Eight further independent breast cancer data sets were located via Oncomine, the GEO database and ArrayExpress [67–69]. All studies considered both ER positive and ER negative breast cancers and were profiled on either Affymetrix or Illumina platforms [33–40]. With the exception of two, all studies were annotated with overall survival data, in the two remaining cases, relapse and distant metastasis were used as proxies for survival. Two studies also lacked ER immunohistochemistry, in these cases, a dip test was utilised to confirm that the distribution of *ESR1* expression was significantly bimodal, before partitioning around medoids was utilised on *ESR1* expression to generate two clusters dividing samples into ER positive and ER negative subgroups. The sample counts, stratified by ER status for all data sets used are provided in **S1 Table**, alongside GEO and ArrayExpress accession numbers. Normalised data from each study was downloaded from the GEO database or ArrayExpress. Quantile normalisation was subsequently performed across all samples within each study.
For our investigation into lung adenocarcinoma we first considered The Cancer Genome Atlas (TCGA) data set of RNAseqv2 data profiling 455 tumours (*http://cancergenome.nih.gov*) and the Director's Challenge microarray data set profiling 398 tumours, with accompanying clinical annotation. Processed data from both studies was quantile normalised and log transformed. The RNAseqv2 data was skewed towards low values in a manner that inhibited the computation of signalling entropy, consequently, genes with read counts below

3 were removed from the data set.
Four further independent lung adenocarcinoma data sets with survival data were located via Oncomine, and the GEO database [42, 45, 46, 67, 68, 70]. Normalised data from each study was downloaded from the GEO database and quantile normalised within each study.
For each study separately probes in the microarrays and sequences in the RNAseqv2 data were matched to unique EntrezGene identifiers; probes or sequences mapping to the same identifier were averaged over.

Construction of the Protein Interaction Network

The Protein Interaction Network (PIN) used in this study was constructed as described in [16]. Briefly, we integrated interaction data obtained from Pathway Commons (*www.pathwaycommons.org*) [71] with the Human Protein Reference Database [72], the National Cancer Institute Nature Pathway Interaction Database (NCI-PID) (*pid.nci.nih.gov*), the Interactome (Intact) (*http://www.ebi.ac.uk/intact/*) and the Molecular Interaction Database (MINT) (*http://mint.bio.uniroma2.it/mint/*). Connections in this network describe a wide array of protein interactions including protein complex formation, post-translational modifications and enzymatic reactions. Notably the PIN includes 20 highly curated immune and cancer signalling pathways from NetPath (*www.netpath.org*) [73]. Redundant interactions were removed from the PIN and only genes with an EntrezGene identifier annotation were retained. The PIN was subsequently sparsified by the consideration of the cellular localisation of interacting proteins to remove interactions likely to by false positives. The resulting maximally connected PIN contained 8,434 nodes and over 300,000 interactions.

Gene Set Enrichment Analysis

Gene Set Enrichment Analysis (GSEA) was implemented using a Fisher's exact test to compare gene lists associated with signalling entropy's prognostic power against the gene sets defined by the Molecular Signatures Database [50, 74]. Computations were performed via software downloaded from the Molecular Signatures Data Base (*www.broadinstitute.org/msigdb*) [50, 74].

Breast Cancer Transcriptomic Signatures

We compared the prognostic power of signalling entropy in breast cancer to two transcriptomic signatures, namely the Embryonic Stem (ES) cell based 100 gene signature of Ben-Porath *et al.* and the 97 gene grade signature of Sotiriou *et al.* These signatures were selected due to their similarity to signalling entropy in their clinical variable association in breast cancer. Like signalling entropy both signatures are prognostic and correlate with the level of differentiation of a tumour. The Ben-Porath *et al.* signature also correlates with pluripotency in healthy tissue and associates with ER status and molecular subtype, further properties of signalling entropy. Given this relatedness, it was important to assess the independence of these signatures from our measure.
All genes in the Ben-Porath *et al.* signature are positively correlated with tumour grade, and thus we computed a score for this signature as the mean log-normalised intensity of these genes in a sample.
The Sotiriou *et al.* signature contains genes both up-regulated and down-regulated in higher grade breast cancers compared to lower grade. We therefore computed a score for this signature as the statistic of a t-test evaluating the hypothesis that the log-normalised intensities of the up-regulated genes is higher than that of the down-regulated genes.

The Signalling Entropy Prognostic Score in Breast Cancer

For the breast cancer data the signalling entropy prognostic score (SE score) was computed from the METABRIC discovery data set from prognostic genes, which were correlated or anti-correlated with signalling entropy independently of grade and ER status, and whose prognostic power was also independent of grade and ER status. This gene set of 320 genes was refined by fitting a Cox proportional hazards model on 5 year censored data, using all the identified genes as covariates and deleting genes which were not significantly prognostic, independently of others in the gene set. This resulted in a small set of 81 genes, 10 of which were negatively correlated with signalling entropy and 71 of which were positively correlated **S2 Table**. A

Signalling Entropy prognostic score (SE score) was then defined as the t-statistic evaluating the hypothesis that the 71 positively correlated genes are expressed more highly than the 10 negatively correlated genes (after z-score normalising the data for each gene, across samples).
Before survival analysis the SE score was normalised by its standard deviation within each study.

The Signalling Entropy Prognostic Score in Lung Adenocarcinoma

For the lung adenocarcinoma data the SE score was computed analogously to the computation of the breast cancer SE score, using the Director's Challenge data set as a discovery set. The only differences were that that adjustment was made for tumour stage rather than grade and ER status and survival analysis was performed on 3 year censored data rather than 5 year. The basis of the score was a small set of 27 genes, 8 of which were negatively correlated with signalling entropy and 19 of which were positively correlated (**S4 Table**). The SE score was then computed as the statistic of the t-test evaluating the hypothesis that the genes negatively correlated with signalling entropy were less expressed than those positively correlated (after z-score normalising each gene, across samples).
Before survival analysis the SE score was normalised by its standard deviation within each study.

MammaPrint and *CADM1* expression

A MammaPrint score was assigned to each sample in the 10 breast cancer datasets and was evaluated from the expression of the 70 genes required to define the signature as the t-statistic comparing the genes found to positively correlate with survival against those negatively correlated with survival in the study of [24].
Before survival analysis the MammaPrint score was normalised by its standard deviation within each study.
CADM1 expression was found in all 6 lung adenocarcinoma data sets and was similarly normalised by its standard deviation within each data set before survival analysis.

OncotypeDX and Kratz et al. score approximations

OncotypeDX and the Kratz *et al.* scores are both derived from RT-PCR rather than from microarrays like MammaPrint and *CADM1* expression. Consequentially, the comparison of the SE score to these scores is not conclusive as we only consider microarray and RNAseq data, which though correlated, is normalised differently to RT-PCR data. Moreover some of the array platforms considered lack expression of certain genes required to compute the OncotypeDX and Kratz *et al.* scores.
We therefore approximate these scores from our data to allow a rough comparison. In the case of OncotypeDX, a score was computed from the expression of 21 genes (or as many as were represented in the data set) in each breast cancer microarray sample using the formula defined by reference [75]. In the case of the Kratz *et al.* score, this was similarly approximated from the expression of 14 genes (or as many as were represented in the data set) in each lung adenocarcinoma microarray or RNAseq sample, using the formula defined by reference [22].

Meta-analysis of prognostic scores

Meta-analysis was performed to combine survival statistics for signalling entropy, the SE score, MammaPrint, *CADM1* expression and the approximations of OncotypeDX and the Kratz *et al.* score. Concordance indices were computed for each prognostic measure in each data set considered and combined using a random effects model. The p-values were combined using Fisher's combined test. Forest plots were generated using the *survcomp* package in R [76].

Evaluation of random gene expression signatures

The 3 random gene expression signatures described by Venet *et al.* were obtained from the supplementary information provided in reference [32]. Following the analysis described by Venet *et al.*, the probes in the METABRIC data sets mapping to genes in each random gene set were extracted, genes which were associated with cell cycling as described by Ben-Porath et al. [12] were removed. The expression values were then median polished and a principal component analysis was performed. Samples were then partitioned into two groups,

for each score via the median PC1 value. A Cox regression on 5 year censored data was then performed to evaluate the prognostic power of each random gene expression signature in each METABRIC data set. Only KRISHNAN2007DEFEAT was significantly prognostic in both METABRIC data sets, however partition of the datasets into ER+ and ER- samples mitigated this prognostic association.

Supplementary Results

Signalling entropy correlates with measures of tumour differentiation in breast cancer

Previously we demonstrated that signalling entropy correlated with differentiation potential in healthy tissue, was elevated in cancerous tissue and was higher in CSCs than in the tumour bulk [16]. We therefore anticipated that signalling entropy would correlate with transcriptomic and histological measures of the level of tumour differentiation.

We consider this hypothesis in the METABRIC breast cancer data sets. Two appropriate transcriptomic measures of tumour differentiation in breast cancer are the Ben-Porath 100 gene Embryonic Stem (ES) cell signature (found to associate with both breast cancer and lung adenocarcinoma tumour grade [12, 13]) and the 97 gene Sotiriou grade signature [31].

As expected, we found that both signatures were strongly correlated with signalling entropy in the METABRIC data sets (Ben-Porath: $p < 2.2e-16$, Sortiriou: $p < 2.2e-16$, **S1 Figure**) indicating that the plastic signalling regime measured by signalling entropy is indeed associated with external, transcriptomic measures of the stemness of a tumour.

As anticipated, signalling entropy also strongly correlated with histological tumour grade in breast cancer, being significantly higher in grade 3 tumours compared with grade 2 and significantly higher in grade 2 tumours as compared with grade 1 ($p < 7.3e-15$ and $p < 2.6e-4$ respectively, **S2A Figure**). This result is consistent with the findings of Ben-Porath *et al.*, which demonstrated that high grade tumours displayed enrichment of an ES cell transcriptomic signature [12]. Importantly, however, we found that unlike signalling entropy the Ben-Porath signature was unable to distinguish between grade 1 and grade 2 tumours in the discovery set of METABRIC (Ben-Porath signature: $p = 0.4$, signalling entropy: $p < 2.6e-4$, **S2B Figure**). This result suggests that our measure is capable of discerning between tumours of varying levels of differentiation, and indeed is more sensitive to such variation than an ES cell transcriptomic signature. We also note that tumours with a high signalling entropy display a bi-modality of enrichment for the Ben-Portah ES cell signature. High ES cell enriched, high signalling entropy tumours are generally grade 3, whereas the low ES cell enriched, high signalling entropy tumours can be of lower grade (**S1 Figure**). This is indicative that a high signalling entropy is related to but not solely determined by the level of differentiation of a tumour, and that other factors, such as inter-cellular heterogeneity may cause high signalling entropy in certain samples.

We also found that signalling entropy correlated with tumour cellularity and was highest in those samples with the greatest proportion of cancerous cells ($p < 7.7e-15$). This result is consistent with our previous finding that cancer cells display a higher level of signalling promiscuity than their healthy counterparts [16,20].

Signalling Entropy correlates with levels of tumour differentiation in lung adenocarinoma

We next considered whether signalling entropy also associated with transcriptomic and histological assessments of tumour differentiation in lung adenocarcinoma. The Ben-Porath signature was investigated by Hassan *et al.* in the context of lung adenocarcinoma and found to show association with histological grade and clinical outcome [13].

By considering the Director's Challenge data set we found that, as in breast cancer, signalling entropy correlated strongly with the Ben-Porath stem cell signature in lung adenocarcinoma ($p < 2.2e-16$, **S5A Figure**). Moreover, we found that signalling entropy correlated strongly with histological assessments of tumour differentiation in lung adenocarcinoma, being highest in poorly differentiated tumours, then moderately differentiated tumours ($p < 1.4e-4$ poorly *vs.* moderately differentiated) and lowest in well differentiated tumours ($p < 2.5e-5$ moderately *vs.* well differentiated, **S5B Figure**). These results further support the notion that signalling entropy is a measure of the stemness of a tumour.

Signalling entropy associates with breast cancer heterogeneity and luminal B breast cancer displays the highest signalling entropy, yet among the weakest enrichment for ES cell genes

We next examined whether signalling entropy was associated with breast cancer subtypes to ascertain whether a stem cell like signalling regime, or inter-cellular diversity could be related to breast cancer heterogeneity as previously suggested [12, 14].

We divided the samples in both the discovery and validation sets of METABRIC by their Pfam50 intrinsic subtype classifications [77] and found that signalling entropy was strongly associated with molecular subtype. Normal tumours displayed the lowest signalling entropy, followed by luminal A tumours (luminal A *vs* normal $p < 2.5e-8$), then HER2 tumours (HER2 *vs* luminal A $p < 1.9e-12$), and lastly luminal B and basal tumours (luminal B *vs* HER2 $p < 0.02$ basal *vs* luminal B $p = 0.3$), which displayed statistically equivalent signalling entropies. This result is in concordance with that of Ben-Porath *et al.* [12], who demonstrated that basal breast cancers displayed a stronger enrichment of an ES cell transcriptomic signature than luminal A breast cancer.

To more fully compare the Ben-Porath signature with signalling entropy, we examined its ability to discriminate between the intrinsic subtypes of breast cancer. As reported in the presenting paper, the signature is most enriched in the basal breast cancer subtype and least in luminal A. However the overall ordering of the intrinsic subtypes by Ben-Porath signature enrichment is quite dissimilar from the ordering by signalling entropy. Notably luminal B breast cancers are significantly less enriched for the Ben-Porath signature than basal ($p < 2.2e-16$), however, signalling entropy suggests that these subtypes share a highly promiscuous signalling regime. This result is intriguing as it implies that the severity of luminal B breast cancer may be linked to a plastic signalling regime, rather than the enrichment of genes typically over-expressed in ES cells. One may conclude from this that the high signalling entropy of the luminal B subtype may be driven by inter-cellular heterogeneity, rather than high intra-cellular signalling promiscuity.

Criticism of molecular subtyping has derived from the lack of diversity in the histological subtype of tumours used to define the classifications [78]. Consequently, we also examined the association between signalling entropy and histological subtype. This revealed that medullary carcinomas have the highest signalling entropy, consistent with these cancers generally being of higher grade and displaying a basal phenotype [79]. Invasive ductal carcinomas of no special type (IDCs-NST) held the second highest signalling entropy, significantly higher than invasive lobular carcinomas ($p < 2.3e-5$) mixed ductal and lobular carcinomas ($p < 1e-3$) and tubular carcinomas, which all displayed a statistically identical signalling entropy. This result suggests that the different histological subtypes display significant differences in the promiscuity of their signalling regimes, a result which may reflect differences in the potency of their cell of origin, or in their degree of de-differentiation from a common originator.

We also found that signalling entropy could discriminate grade matched ER positive tumours from ER negative tumours ($p < 0.01$). Given the finding by Ben-Porath *et al.* that ER negative tumours were enriched for an ES cell signature [12], this result suggests that a stem cell-like, promiscuous signalling regime is more prevalent in ER negative tumours, regardless of histological grade.

Tumours carrying a mutation in p53 have also been demonstrated to be enriched for an ES cell gene expression signature [80]. In line with this result we found that grade matched p53 mutated tumours displayed a higher signalling entropy as compared their wild type counterparts ($p = 0.005$). This result is consistent with the hypothesis that p53 mutations in breast cancer can facilitate de-differentiation [80].

Signalling Entropy associates with smoking history and tumour stage in lung adenocarcinoma

We next investigated whether exposure to cigarette smoke was associated with a plastic signalling regime in lung adenocarcinoma. Hassan *et al.*, previously reported that lung adenocarcinoma patients with a positive smoking history displayed an increased expression of stem cell genes. In line with these findings, we found a strong association between signalling entropy and smoking history in lung adenocarcinoma (TCGA adenocarcinoma: never smoked *vs.* currently smoking $p = 1.4e-7$, Director's Challenge adenocarcinoma: never smoked *vs.* currently smoking $p < 0.03$, **S6 Figure**). These results suggest that cigarette smoking

induces an increased signalling promiscuity in lung adenocarcinoma.
We also found that signalling entropy significantly correlated with tumour stage in both the TCGA and Director's Challenge lung adenocarcinoma data sets ($p < 4.8e-8$), suggesting that signalling entropy associates with current leading assessments of prognosis.

Signalling entropy is elevated in heterogeneous samples on average

Preliminaries

Let $\mathcal{G} = (V, E)$ be an undirected graph, where $V = \{v_1, \ldots, v_n\}$ is a set of vertices and $E = \{(i, j) | i, j \in V\}$ a set of edges; we denote the adjacency matrix of $\mathcal{G}$ by $A = (a_{ij})_{ij \in V}$. In our analysis $\mathcal{G}$ represents the undirected topology of the interactome.
To each vertex $i \in V$ we assign a variable $x_i \in \mathbb{R}^{>0}$, and denote the vector containing all such variables by $x = (x_i)_{i=1}^n \in \Omega \subset \mathbb{R}^{>0}$, where Ω is some bounded domain. In our analysis x will represent the vector of log normalised gene expression values for a homogeneous sample, we note that as the expression of genes cannot be infinite we bound x within a finite domain Ω.
We consider a random walk, on the graph $\mathcal{G}$; with transition probability matrix $P(x) = (p_{ij}(x))_{ij \in V}$ defined via

$$p_{ij}(x) = \frac{a_{ij} x_j}{\sum_{k \in V} a_{ik} x_k}.$$

We define the following measures

1. The local entropy of vertex $i \in V$, defined by

$$S_i(x) := -\sum_{j \in V} p_{ij}(x) \log p_{ij}(x). \tag{1}$$

2. The entropy rate of $P(x)$, defined by

$$S_R(x) := \sum_{i \in V} \pi_i(x) S_i(x). \tag{2}$$

where π_i denotes the stationary distribution of $P(x)$ and satisfies

$$\pi_j(x) = \sum_{i \in V} p_{ij}(x) \pi_i(x). \tag{3}$$

We will refer to $S_R(x)$ as the signalling entropy of x, as it is equivalent up to a normalisation factor.

Let us also define the following functions

$$W_{ij}(x) := a_{ij} x_i x_j$$

$$W_i(x) := \sum_j W_{ij}(x)$$

$$W(x) := \sum_i W_i(x).$$

We also note that by multiplying $p_{ij}(x)$ by $1 = x_i / x_i$, we see that

$$p_{ij} = \frac{W_{ij}(x)}{W_i(x)}.$$

Thus $P(x)$ describes a weighted random walk on an undirected graph and it thus follows that

$$\pi_i(x) = W_i(x) / W(x).$$

The proof follows from simple substitution into (3):

$$\begin{aligned} LHS &= \sum_i p_{ij}(x)\pi_i(x) \\ &= \sum_i \frac{W_{ij}(x)}{W_i(x)}\frac{W_i(x)}{W(x)} \\ &= \frac{W_j(x)}{W(x)} \\ &= RHS. \end{aligned}$$

From this result it follows that

$$S_R(x) = -\frac{1}{W(x)}\sum_{ij} W_{ij}(x)\log\frac{W_{ij}(x)}{W_i(x)} \tag{4}$$

Motivation

In this section we wish to demonstrate that the signalling entropy of a heterogeneous sample generated from a 50:50 mixture of two cell types is greater, on average, than the signalling entropy of a homogeneous sample. This amounts to proving the following proposition:

Proposition. *Let $x, y \in \Omega$, then*

$$\int_\Omega\int_\Omega \left(S_R\left(\frac{x+y}{2}\right) - S_R(x)\right) dxdy > 0. \tag{5}$$

Let us consider the following claim

Claim (Super-additivity)**.** *Let $x, y \in \Omega$ then*

$$S_R\left(\frac{x+y}{2}\right) > \frac{S_R(x)}{2} + \frac{S_R(y)}{2}. \tag{6}$$

It is clear that if the claim is true then the proposition must be true. Notice first that if the claim is true then as it is a strict bound $\exists\epsilon > 0$ such that $S_R\left(\frac{x+y}{2}\right) > \frac{S_R(x)}{2} + \frac{S_R(y)}{2} + \epsilon$. Whence

$$\begin{aligned} \int_\Omega\int_\Omega \left(S_R\left(\frac{x+y}{2}\right) - S_R(x)\right) dxdy &> \int_\Omega\int_\Omega \left(\frac{S_R(y)}{2} - \frac{S_R(x)}{2} + \epsilon\right) dxdy & (7) \\ &= |\Omega|^2\epsilon & (8) \\ &> 0, & (9) \end{aligned}$$

and thus the proposition is true.
We will therefore derive in a sufficient condition for the claim to be true. We will then demonstrate numerically that this condition holds for over 528 distinct in silico mixtures of homogeneous healthy differentiated tissues.
The results in this section thus provide evidence that signalling entropy is raised in heterogeneous biological samples comprising of a 50:50 mix of two cell types, as compared to homogeneous samples, at the population level.

A sufficient condition for the claim to be true

Here we will prove the following theorem

Theorem. *Let $x, y \in \Omega$, let $a = \max_i\left(\frac{x_i}{y_i}\right)$, and let $b = \min_i\left(\frac{x_i}{y_i}\right)$. A sufficient condition for the claim above to be true is*

$$sign(1 - 1/b + 2/a) + sign(1 - a + 2b) = 2 \tag{10}$$

Proof: From (4)

$$S_R((x+y)/2) = -\frac{1}{W((x+y)/2)} \sum_{ij} W_{ij}((x+y)/2) \log \frac{W_{ij}((x+y)/2)}{W_i((x+y)/2)}, \tag{11}$$

it is therefore prudent to first consider $W_{ij}((x+y)/2)$:

$$\begin{aligned} W_{ij}((x+y)/2) &= \frac{a_{ij}}{4}(x_i+y_i)(x_j+y_j) \quad (12) \\ &= \frac{1}{4}(W_{ij}(x)+W_{ij}(y)+a_{ij}(x_iy_j+x_jy_i)). \quad (13) \end{aligned}$$

We will define

$$\hat{W}_{ij}(x,y) = a_{ij}(x_iy_j + x_jy_i) \tag{14}$$

$$\hat{W}_i(x,y) = \sum_j \hat{W}_{ij}(x,y) \tag{15}$$

$$\hat{W}(x,y) = \sum_j \hat{W}_i(x,y) \tag{16}$$

for notational ease. Note that as $y, x > 0$

$$\hat{W}_{ij}(x,y) = \frac{x_i}{y_i} W_{ij}(y) + \frac{y_i}{x_i} W_{ij}(x). \tag{17}$$

$$\hat{W}_i(x,y) = \frac{x_i}{y_i} W_i(y) + \frac{y_i}{x_i} W_i(x). \tag{18}$$

(19)

It thus follows that:

$$S_R((x+y)/2) = \frac{-1}{W(x)+W(y)+\hat{W}(x,y)} \sum_{ij} (W_{ij}(x)+W_{ij}(y)+\hat{W}_{ij}(x,y)) \log \frac{W_{ij}(x)+W_{ij}(y)+\hat{W}_{ij}(x,y)}{W_i(x)+W_i(y)+\hat{W}_i(x,y)}. \tag{20}$$

We will now appeal to the log sum inequality:

Theorem (Log-sum inequality)**.** *Let $a_1, ..., a_n$, $b_1, ..., b_n$ be non-negative numbers then*

$$\sum_{i=1}^n a_i log \frac{a_i}{b_i} \geq \left(\sum_{i=1}^n a_i \right) log \frac{\sum_{i=1}^n a_i}{\sum_{i=1}^n b_i}. \tag{21}$$

If we denote $a_1 = W_{ij}(x), a_2 = W_{ij}(y), a_3 = \hat{W}_{ij}(x,y)$ and $b_1 = W_i(x), b_2 = W_i(y), b_3 = \hat{W}_i(x,y)$, and apply the log-sum inequality to the summand of (22) we obtain:

$$\begin{aligned} S_R((x+y)/2) &\geq \frac{-1}{W(x)+W(y)+\hat{W}(x,y)} \sum_{ij} W_{ij}(x) \log \frac{W_{ij}(x)}{W_i(x)} + \quad (22) \\ & \quad W_{ij}(y) \log \frac{W_{ij}(y)}{W_i(y)} + \hat{W}_{ij}(x,y) \log \frac{\hat{W}_{ij}(x,y)}{\hat{W}_i(x,y)} \quad (23) \\ &= \frac{W(x)S_R(x) + W(y)S_R(y) - \sum_{ij} \hat{W}_{ij}(x,y) \log \frac{\hat{W}_{ij}(x,y)}{\hat{W}_i(x,y)}}{W(x)+W(y)+\hat{W}(x,y)}. \quad (24) \end{aligned}$$

Now consider the term $-\sum_{ij} \hat{W}_{ij}(x,y) \log \frac{\hat{W}_{ij}(x,y)}{\hat{W}_i(x,y)}$ and apply the log sum inequality again:

$$\begin{aligned} -\sum_{ij} \hat{W}_{ij}(x,y) \log \frac{\hat{W}_{ij}(x,y)}{\hat{W}_i(x,y)} &= -\sum_{ij} \left(\frac{x_i}{y_i} W_{ij}(y) + \frac{y_i}{x_i} W_{ij}(x) \right) \log \frac{\frac{x_i}{y_i} W_{ij}(y) + \frac{y_i}{x_i} W_{ij}(x)}{\frac{x_i}{y_i} W_i(y) + \frac{y_i}{x_i} W_i(x)} \\ &\geq -\sum_{ij} \frac{x_i}{y_i} W_{ij}(y) \log \frac{W_{ij}(y)}{W_i(y)} + \frac{y_i}{x_i} W_{ij}(x) \log \frac{W_{ij}(x)}{W_i(x)}. \quad (25) \end{aligned}$$

Returning to (22), it now follows that

$$S_R\left(\frac{x+y}{2}\right) - \frac{S_R(x)-S_R(y)}{2} \geq$$
$$\frac{1}{2(W(x)+W(y)+\hat{W}(x,y))}\Bigg(S_R(x)(W(x)-W(y)-\hat{W}(xy)) + S_R(x)(W(y)-W(x)-\hat{W}(xy)) - 2\sum_{ij}\frac{x_i}{y_i}W_{ij}(y)\log\frac{W_{ij}(y)}{W_i(y)} + \frac{y_i}{x_i}W_{ij}(x)\log\frac{W_{ij}(x)}{W_i(x)}\Bigg). \quad (26)$$

So the claim is true if

$$0 < \frac{1}{2(W(x)+W(y)+\hat{W}(x,y))}\Bigg(S_R(x)(W(x)-W(y)-\hat{W}(xy)) + S_R(x)(W(y)-W(x)-\hat{W}(xy)) - 2\sum_{ij}\frac{x_i}{y_i}W_{ij}(y)\log\frac{W_{ij}(y)}{W_i(y)} + \frac{y_i}{x_i}W_{ij}(x)\log\frac{W_{ij}(x)}{W_i(x)}\Bigg). \quad (27)$$

We note that if (27) holds then

$$2\left(W(x)S_R(x)+W(y)S_R(y) - \sum_{ij}\frac{x_i}{y_i}W_{ij}(y)\log\frac{W_{ij}(y)}{W_i(y)} + \frac{y_i}{x_i}W_{ij}(x)\log\frac{W_{ij}(x)}{W_i(x)}\right) > (S_R(x)+S_R(y))(W(x)+W(y)+\hat{W}(xy)) \quad (28)$$

We note that RHS of (28) satisfies

$$2(W(x)S_R(x)+W(y)S_R(y) - \sum_{ij}\frac{x_i}{y_i}W_{ij}(y)\log\frac{W_{ij}(y)}{W_i(y)} + \frac{y_i}{x_i}W_{ij}(x)\log\frac{W_{ij}(x)}{W_i(x)} > 2W(x)S_R(x)(1+\min_i\frac{y_i}{x_i}) + 2W(y)S_R(y)(1+\min_i\frac{x_i}{y_i}) \quad (29)$$

We notice that

$$\hat{W}(xy) = \sum_i \frac{x_i}{y_i}W_i(y) + \frac{y_i}{x_i}W_i(x) \quad (30)$$
$$< \max_i\frac{x_i}{y_i}W(y) + \max_i\frac{y_i}{x_i}W(x) \quad (31)$$

whence the LHS of (28) satisfies

$$(S_R(x)+S_R(y))(W(x)+W(y)+\hat{W}(xy)) < S_R(x)W(x)(1+\max_i\frac{y_i}{x_i}) + S_R(y)W(y)(1+\max_i\frac{x_i}{y_i}). \quad (32)$$

Whence it follows that if

$$S_R(x)W(x)(1+\max_i\frac{y_i}{x_i}) + S_R(y)W(y)(1+\max_i\frac{x_i}{y_i}) < 2W(x)S_R(x)(1+\min_i\frac{y_i}{x_i}) + 2W(y)S_R(y)(1+\min_i\frac{x_i}{y_i}) \quad (33)$$

$$S_R(x)W(x)(1-\max_i\frac{y_i}{x_i}+2\min_i\frac{y_i}{x_i}) + S_R(y)W(y)(1-\max_i\frac{x_i}{y_i}+2\min_i\frac{x_i}{y_i}) > 0 \quad (34)$$

$$S_R(x)W(x)(1-1/b+2/a) + S_R(y)W(y)(1-a+2b) > 0 \quad (35)$$

then LHS<RHS. Where $a = \max_i \left(\frac{x_i}{y_i}\right)$, and $b = \min_i \left(\frac{x_i}{y_i}\right)$, as in the claim. We note that as $S_R(x)W(x) > 0$ and $S_R(y)W(y) > 0$ than the condition will always hold provided

$$sign(1 - 1/b + 2/a) + sign(1 - a + 2b) = 2.$$

Hence the theorem is correct.

□

We note that the condition can be computed numerically for a range of values and holds for the majority of biologically plausible ranges (**S8 Figure**).

Empirical validation that the claim is true on Ω

If the condition derived above holds in Ω, the space of biologically admissible homogeneous sample expression regimes, then the claim and hence the proposition explained at the start of this section are true and we have proven our postulate.
To investigate whether this is the case we consider the data set described in GSE2361 [29], which profiles 33 distinct adult tissues and 3 foetal tissues. We will disregard the foetal tissues as the tissue types overlap with others adult tissues and thus cannot be considered distinct homogeneous samples.
We first computed the value of $sign(1 - 1/b + 2/a) + sign(1 - a + 2b)$ for every pairwise combination of the 33 tissue types (528 pairwise combinations). We found that for every combination the condition $sign(1 - 1/b + 2/a) + sign(1 - a + 2b) = 2$ was satisfied and thus the claim was correct for this data set (**S9 Figure**). While it is certainly true that not every tissue, when mixed with another, will display an increased signalling entropy, we do see a trend towards this happening, with the reproductive tissues (testes, uterus, ovaries, breast) providing the most significant increases (**S10 Figure**).
Finally, as expected, the proposition is indeed correct for this data set, and the signalling entropy of the mixed samples is higher than the homogeneous samples on average; this increase is also significant $p = 0.012$ (paired Wilcoxon test) (**S11 Figure**).

Summary

To conclude, we have derived a condition which if satisfied by homogeneous samples guarantees that signalling entropy will be higher in heterogeneous 50:50 mixtures of two homogeneous samples, as compared to the unmixed samples, on average. We then verified that for a large number of homogeneous adult tissues the condition was indeed satisfied and signalling entropy was higher in 50:50 mixtures of homogeneous tissues on average.
These result suggest signalling entropy represents a quantifier of intra-sample sample heterogeneity, being correlated with heterogeneity at the population level.

References

1. Reya T, Morrison SJ, Clarke MF, Weissman IL (2001) Stem cells, cancer, and cancer stem cells. Nature 414: 105-11.

2. Stingl J, Caldas C (2007) Molecular heterogeneity of breast carcinomas and the cancer stem cell hypothesis. Nat Rev Cancer 7: 791-9.

3. Shackleton M, Quintana E, Fearon ER, Morrison SJ (2009) Heterogeneity in cancer: cancer stem cells versus clonal evolution. Cell 138: 822-9.

4. Nowell PC (1976) The clonal evolution of tumor cell populations. Science 194: 23-8.

5. Heppner GH (1984) Tumor heterogeneity. Cancer Res 44: 2259-65.

6. Fidler IJ, Hart IR (1982) Biological diversity in metastatic neoplasms: origins and implications. Science 217: 998-1003.

7. Al-Hajj M, Wicha MS, Benito-Hernandez A, Morrison SJ, Clarke MF (2003) Prospective identification of tumorigenic breast cancer cells. Proc Natl Acad Sci U S A 100: 3983-8.

8. Pinto CA, Widodo E, Waltham M, Thompson EW (2013) Breast cancer stem cells and epithelial mesenchymal plasticity - implications for chemoresistance. Cancer Lett 341: 56-62.

9. Creighton CJ, Li X, Landis M, Dixon JM, Neumeister VM, Sjolund A, et al. (2009) Residual breast cancers after conventional therapy display mesenchymal as well as tumor-initiating features. Proc Natl Acad Sci U S A 106: 13820-5.

10. de Beca FF, Caetano P, Gerhard R, Alvarenga CA, Gomes M, Paredes J, et al. (2013) Cancer stem cells markers CD44, CD24 and ALDH1 in breast cancer special histological types. J Clin Pathol 66: 187-91.

11. Bruna A, Greenwood W, Le Quesne J, Teschendorff A, Miranda-Saavedra D, Rueda OM, et al. (2012) TGF-beta induces the formation of tumour-initiating cells in claudinlow breast cancer. Nat Commun 3: 1055.

12. Ben-Porath I, Thomson MW, Carey VJ, Ge R, Bell GW, Regev A, et al. (2008) An embryonic stem cell-like gene expression signature in poorly differentiated aggressive human tumors. Nat Genet 40: 499-507.

13. Hassan KA, Chen G, Kalemkerian GP, Wicha MS, Beer DG (2009) An embryonic stem cell-like signature identifies poorly differentiated lung adenocarcinoma but not squamous cell carcinoma. Clin Cancer Res 15: 6386-90.

14. Prat A, Parker JS, Karginova O, Fan C, Livasy C, Herschkowitz JI, et al. (2010) Phenotypic and molecular characterization of the claudin-low intrinsic subtype of breast cancer. Breast Cancer Res 12: R68.

15. Taube JH, Herschkowitz JI, Komurov K, Zhou AY, Gupta S, Yang J, et al. (2010) Core epithelial-to-mesenchymal transition interactome gene-expression signature is associated with claudin-low and metaplastic breast cancer subtypes. Proc Natl Acad Sci U S A 107: 15449-54.

16. Banerji CRS, Miranda-Saavedra D, Severini S, Widschwendter M, Enver T, Zhou JX, et al. (2013) Cellular network entropy as the energy potential in waddington's differentiation landscape. Sci Rep 3: 3039.

17. Gerlinger M, Rowan AJ, Horswell S, Larkin J, Endesfelder D, Gronroos E, et al. (2012) Intratumor heterogeneity and branched evolution revealed by multiregion sequencing. N Engl J Med 366: 883-92.

18. Maley CC, Galipeau PC, Finley JC, Wongsurawat VJ, Li X, Sanchez CA, et al. (2006) Genetic clonal diversity predicts progression to esophageal adenocarcinoma. Nat Genet 38: 468-73.

19. Vogelstein B, Papadopoulos N, Velculescu VE, Zhou S, Diaz J L A, Kinzler KW (2013) Cancer genome landscapes. Science 339: 1546-58.

20. West J, Bianconi G, Severini S, Teschendorff AE (2012) Differential network entropy reveals cancer system hallmarks. Sci Rep 2: 802.

21. Siegel R, Naishadham D, Jemal A (2013) Cancer statistics, 2013. CA Cancer J Clin 63: 11-30.

22. Kratz JR, He J, Van Den Eeden SK, Zhu ZH, Gao W, Pham PT, et al. (2012) A practical molecular assay to predict survival in resected non-squamous, non-small-cell lung cancer: development and international validation studies. Lancet 379: 823-32.

23. Bergot E, Levallet G, Campbell K, Dubois F, Lechapt E, Zalcman G (2013) Predictive biomarkers in patients with resected non-small cell lung cancer treated with perioperative chemotherapy. Eur Respir Rev 22: 565-76.

24. van 't Veer LJ, Dai H, van de Vijver MJ, He YD, Hart AA, Mao M, et al. (2002) Gene expression profiling predicts clinical outcome of breast cancer. Nature 415: 530-6.

25. van de Vijver MJ, He YD, van't Veer LJ, Dai H, Hart AA, Voskuil DW, et al. (2002) A gene-expression signature as a predictor of survival in breast cancer. N Engl J Med 347: 1999-2009.

26. Andre F, Delaloge S (2010) First-generation genomic tests for breast cancer treatment. Lancet Oncol 11: 6-7.

27. Cardoso F, Piccart-Gebhart M, Van't Veer L, Rutgers E (2007) The mindact trial: the first prospective clinical validation of a genomic tool. Mol Oncol 1: 246-51.

28. van Wieringen WN, van der Vaart AW (2011) Statistical analysis of the cancer cell's molecular entropy using high-throughput data. Bioinformatics 27: 556-63.

29. Ge X, Yamamoto S, Tsutsumi S, Midorikawa Y, Ihara S, Wang SM, et al. (2005) Interpreting expression profiles of cancers by genome-wide survey of breadth of expression in normal tissues. Genomics 86: 127-41.

30. Curtis C, Shah SP, Chin SF, Turashvili G, Rueda OM, Dunning MJ, et al. (2012) The genomic and transcriptomic architecture of 2,000 breast tumours reveals novel subgroups. Nature 486: 346-52.

31. Sotiriou C, Wirapati P, Loi S, Harris A, Fox S, Smeds J, et al. (2006) Gene expression profiling in breast cancer: understanding the molecular basis of histologic grade to improve prognosis. J Natl Cancer Inst 98: 262-72.

32. Venet D, Dumont JE, Detours V (2011) Most random gene expression signatures are significantly associated with breast cancer outcome. PLoS Comput Biol 7: e1002240.

33. Miller LD, Smeds J, George J, Vega VB, Vergara L, Ploner A, et al. (2005) An expression signature for p53 status in human breast cancer predicts mutation status, transcriptional effects, and patient survival. Proc Natl Acad Sci U S A 102: 13550-5.

34. Pawitan Y, Bjohle J, Amler L, Borg AL, Egyhazi S, Hall P, et al. (2005) Gene expression profiling spares early breast cancer patients from adjuvant therapy: derived and validated in two population-based cohorts. Breast Cancer Res 7: R953-64.

35. Desmedt C, Piette F, Loi S, Wang Y, Lallemand F, Haibe-Kains B, et al. (2007) Strong time dependence of the 76-gene prognostic signature for node-negative breast cancer patients in the transbig multicenter independent validation series. Clin Cancer Res 13: 3207-14.

36. Kao KJ, Chang KM, Hsu HC, Huang AT (2011) Correlation of microarray-based breast cancer molecular subtypes and clinical outcomes: implications for treatment optimization. BMC Cancer 11: 143.

37. Loi S, Haibe-Kains B, Desmedt C, Lallemand F, Tutt AM, Gillet C, et al. (2007) Definition of clinically distinct molecular subtypes in estrogen receptor-positive breast carcinomas through genomic grade. J Clin Oncol 25: 1239-46.

38. Wang Y, Klijn JG, Zhang Y, Sieuwerts AM, Look MP, Yang F, et al. (2005) Gene-expression profiles to predict distant metastasis of lymph-node-negative primary breast cancer. Lancet 365: 671-9.

39. Chin K, DeVries S, Fridlyand J, Spellman PT, Roydasgupta R, Kuo WL, et al. (2006) Genomic and transcriptional aberrations linked to breast cancer pathophysiologies. Cancer Cell 10: 529-41.

40. Schmidt M, Bohm D, von Torne C, Steiner E, Puhl A, Pilch H, et al. (2008) The humoral immune system has a key prognostic impact in node-negative breast cancer. Cancer Res 68: 5405-13.

41. Viale G, Slaets L, Bogaerts J, Rutgers E, van't Veer L, Piccart-Gebhart MJ, et al. (2014) High concordance of protein (by ihc), gene (by fish; her2 only), and microarray readout (by targetprint) of er, pgr, and her2: results from the eortc 10041/big 03-04 mindact trial. Ann Oncol 25: 816-23.

42. Shedden K, Taylor JM, Enkemann SA, Tsao MS, Yeatman TJ, Gerald WL, et al. (2008) Gene expression-based survival prediction in lung adenocarcinoma: a multi-site, blinded validation study. Nat Med 14: 822-7.

43. Yamauchi M, Yamaguchi R, Nakata A, Kohno T, Nagasaki M, Shimamura T, et al. (2012) Epidermal growth factor receptor tyrosine kinase defines critical prognostic genes of stage i lung adenocarcinoma. PLoS One 7: e43923.

44. Botling J, Edlund K, Lohr M, Hellwig B, Holmberg L, Lambe M, et al. (2013) Biomarker discovery in non-small cell lung cancer: integrating gene expression profiling, meta-analysis, and tissue microarray validation. Clin Cancer Res 19: 194-204.

45. Der SD, Sykes J, Pintilie M, Zhu CQ, Strumpf D, Liu N, et al. (2014) Validation of a histology-independent prognostic gene signature for early-stage, non-small-cell lung cancer including stage ia patients. J Thorac Oncol 9: 59-64.

46. Sato M, Larsen JE, Lee W, Sun H, Shames DS, Dalvi MP, et al. (2013) Human lung epithelial cells progressed to malignancy through specific oncogenic manipulations. Mol Cancer Res 11: 638-50.

47. Ein-Dor L, Kela I, Getz G, Givol D, Domany E (2005) Outcome signature genes in breast cancer: is there a unique set? Bioinformatics 21: 171-8.

48. Michiels S, Koscielny S, Hill C (2005) Prediction of cancer outcome with microarrays: a multiple random validation strategy. Lancet 365: 488-92.

49. Subramanian J, Simon R (2010) Gene expression-based prognostic signatures in lung cancer: ready for clinical use? J Natl Cancer Inst 102: 464-74.

50. Liberzon A, Subramanian A, Pinchback R, Thorvaldsdottir H, Tamayo P, Mesirov JP (2011) Molecular signatures database (MSigDB) 3.0. Bioinformatics 27: 1739-40.

51. Kleer CG, Cao Q, Varambally S, Shen R, Ota I, Tomlins SA, et al. (2003) EZH2 is a marker of aggressive breast cancer and promotes neoplastic transformation of breast epithelial cells. Proc Natl Acad Sci U S A 100: 11606-11.

52. Gonzalez ME, Moore HM, Li X, Toy KA, Huang W, Sabel MS, et al. (2014) EZH2 expands breast stem cells through activation of NOTCH1 signaling. Proc Natl Acad Sci U S A 111: 3098-103.

53. Takawa M, Masuda K, Kunizaki M, Daigo Y, Takagi K, Iwai Y, et al. (2011) Validation of the histone methyltransferase ezh2 as a therapeutic target for various types of human cancer and as a prognostic marker. Cancer Sci 102: 1298-305.

54. Shao C, Sullivan JP, Girard L, Augustyn A, Yenerall P, Rodriguez-Canales J, et al. (2014) Essential role of aldehyde dehydrogenase 1A3 for the maintenance of non-small cell lung cancer stem cells is associated with the STAT3 pathway. Clin Cancer Res 20: 4154-66.

55. Takahashi-Yanaga F, Kahn M (2010) Targeting Wnt signaling: can we safely eradicate cancer stem cells? Clin Cancer Res 16: 3153-62.

56. Buijs JT, van der Horst G, van den Hoogen C, Cheung H, de Rooij B, Kroon J, et al. (2012) The BMP2/7 heterodimer inhibits the human breast cancer stem cell subpopulation and bone metastases formation. Oncogene 31: 2164-74.

57. Marjanovic ND, Weinberg RA, Chaffer CL (2013) Cell plasticity and heterogeneity in cancer. Clin Chem 59: 168-79.

58. Chaffer CL, Marjanovic ND, Lee T, Bell G, Kleer CG, Reinhardt F, et al. (2013) Poised chromatin at the zeb1 promoter enables breast cancer cell plasticity and enhances tumorigenicity. Cell 154: 61-74.

59. Bedard PL, Hansen AR, Ratain MJ, Siu LL (2013) Tumour heterogeneity in the clinic. Nature 501: 355-64.

60. Xie Y, Minna JD (2012) A lung cancer molecular prognostic test ready for prime time. Lancet 379: 785-7.

61. Bilal E, Dutkowski J, Guinney J, Jang IS, Logsdon BA, Pandey G, et al. (2013) Improving breast cancer survival analysis through competition-based multidimensional modeling. PLoS Comput Biol 9: e1003047.

62. Dowsett M, Sestak I, Lopez-Knowles E, Sidhu K, Dunbier AK, Cowens JW, et al. (2013) Comparison of pam50 risk of recurrence score with oncotype dx and ihc4 for predicting risk of distant recurrence after endocrine therapy. J Clin Oncol 31: 2783-90.

63. Cuzick J, Dowsett M, Pineda S, Wale C, Salter J, Quinn E, et al. (2011) Prognostic value of a combined estrogen receptor, progesterone receptor, ki-67, and human epidermal growth factor receptor 2 immunohistochemical score and comparison with the genomic health recurrence score in early breast cancer. J Clin Oncol 29: 4273-8.

64. Parker JS, Mullins M, Cheang MC, Leung S, Voduc D, Vickery T, et al. (2009) Supervised risk predictor of breast cancer based on intrinsic subtypes. J Clin Oncol 27: 1160-7.

65. Grimmett G, Stirzaker D (1992) Probability and random processes. Oxford University Press.

66. Demetrius L, Manke T (2005) Robustness and network evolution-an entropic principle. Physica A: Statistical Mechanics and its Applications 346: 682-696.

67. Rhodes DR, Kalyana-Sundaram S, Mahavisno V, Varambally R, Yu J, Briggs BB, et al. (2007) Oncomine 3.0: genes, pathways, and networks in a collection of 18,000 cancer gene expression profiles. Neoplasia 9: 166-80.

68. Barrett T, Wilhite SE, Ledoux P, Evangelista C, Kim IF, Tomashevsky M, et al. (2013) Ncbi geo: archive for functional genomics data sets–update. Nucleic Acids Res 41: D991-5.

69. Rustici G, Kolesnikov N, Brandizi M, Burdett T, Dylag M, Emam I, et al. (2013) Arrayexpress update–trends in database growth and links to data analysis tools. Nucleic Acids Res 41: D987-90.

70. Raponi M, Zhang Y, Yu J, Chen G, Lee G, Taylor JM, et al. (2006) Gene expression signatures for predicting prognosis of squamous cell and adenocarcinomas of the lung. Cancer Res 66: 7466-72.

71. Cerami EG, Gross BE, Demir E, Rodchenkov I, Babur O, Anwar N, et al. (2011) Pathway commons, a web resource for biological pathway data. Nucleic Acids Res 39: D685-90.

72. Prasad TS, Kandasamy K, Pandey A (2009) Human protein reference database and human proteinpedia as discovery tools for systems biology. Methods Mol Biol 577: 67-79.

73. Kandasamy K, Mohan SS, Raju R, Keerthikumar S, Kumar GS, Venugopal AK, et al. (2010) Netpath: a public resource of curated signal transduction pathways. Genome Biol 11: R3.

74. Subramanian A, Tamayo P, Mootha VK, Mukherjee S, Ebert BL, Gillette MA, et al. (2005) Gene set enrichment analysis: a knowledge-based approach for interpreting genome-wide expression profiles. Proc Natl Acad Sci U S A 102: 15545-50.

75. Paik S, Shak S, Tang G, Kim C, Baker J, Cronin M, et al. (2004) A multigene assay to predict recurrence of tamoxifen-treated, node-negative breast cancer. N Engl J Med 351: 2817-26.

76. Haibe-Kains B, Desmedt C, Sotiriou C, Bontempi G (2008) A comparative study of survival models for breast cancer prognostication on microarray data: does a single gene beat them all? Bioinformatics 24(19): 2200-2208.

77. Perou CM, Sorlie T, Eisen MB, van de Rijn M, Jeffrey SS, Rees CA, et al. (2000) Molecular portraits of human breast tumours. Nature 406: 747-52.

78. Weigelt B, Reis-Filho JS (2009) Histological and molecular types of breast cancer: is there a unifying taxonomy? Nat Rev Clin Oncol 6: 718-30.

79. Weigelt, B., Horlings, H. M., Kreike, B., Hayes, M. M., Hauptmann, M., Wessels, L. F. rt al. (2008) Refinement of breast cancer classification by molecular characterization of histological special types J Pathol 216: 141-50.

80. Mizuno H, Spike BT, Wahl GM, Levine AJ (2010) Inactivation of p53 in breast cancers correlates with stem cell transcriptional signatures. Proc Natl Acad Sci U S A 107: 22745-50.
